# Dynamic proteomic profiling of human periodontal ligament stem cells during osteogenic differentiation

**DOI:** 10.1186/s13287-020-02123-6

**Published:** 2021-02-03

**Authors:** Jianjia Li, Zhifa Wang, Xiangyu Huang, Zhaodan Wang, Zehao Chen, Runting Wang, Zhao Chen, Wei Liu, Buling Wu, Fuchun Fang, Wei Qiu

**Affiliations:** 1 Department of Stomatology, Nanfang Hospital, Southern Medical University, 1838 Guangzhou Avenue North, Guangzhou, 510515 China; 2 School of Stomatology, Southern Medical University, 1838 Guangzhou Avenue North, Guangzhou, 510515 China; 3 Department of Stomatology, General Hospital of Southern Theater of PLA, 111 Liuhua Road, Guangzhou, 510010 China; 4 Shenzhen Stomatology Hospital (Pingshan), Southern Medical University, 143 Dongzong Road, Pingshan District, Shenzhen, 518118 China

**Keywords:** Human periodontal ligament stem cell, Osteogenic differentiation, Dynamic proteomics, Oxidative phosphorylation, SOD2

## Abstract

**Background:**

Human periodontal ligament stem cells (hPDLSCs) are ideal seed cells for periodontal regeneration. A greater understanding of the dynamic protein profiles during osteogenic differentiation contributed to the improvement of periodontal regeneration tissue engineering.

**Methods:**

Tandem Mass Tag quantitative proteomics was utilized to reveal the temporal protein expression pattern during osteogenic differentiation of hPDLSCs on days 0, 3, 7 and 14. Differentially expressed proteins (DEPs) were clustered and functional annotated by Gene Ontology (GO) terms. Pathway enrichment analysis was performed based on the Kyoto Encyclopedia of Genes and Genomes database, followed by the predicted activation using Ingenuity Pathway Analysis software. Interaction networks of redox-sensitive signalling pathways and oxidative phosphorylation (OXPHOS) were conducted and the hub protein SOD2 was validated with western blotting.

**Results:**

A total of 1024 DEPs were identified and clustered in 5 distinctive clusters representing dynamic tendencies. The GO enrichment results indicated that proteins with different tendencies show different functions. Pathway enrichment analysis found that OXPHOS was significantly involved, which further predicted continuous activation. Redox-sensitive signalling pathways with dynamic activation status showed associations with OXPHOS to various degrees, especially the sirtuin signalling pathway. SOD2, an important component of the sirtuin pathway, displays a persistent increase during osteogenesis. Data are available via ProteomeXchange with identifier PXD020908.

**Conclusion:**

This is the first in-depth dynamic proteomic analysis of osteogenic differentiation of hPDLSCs. It demonstrated a dynamic regulatory mechanism of hPDLSC osteogenesis and might provide a new perspective for research on periodontal regeneration.

**Graphical abstract:**

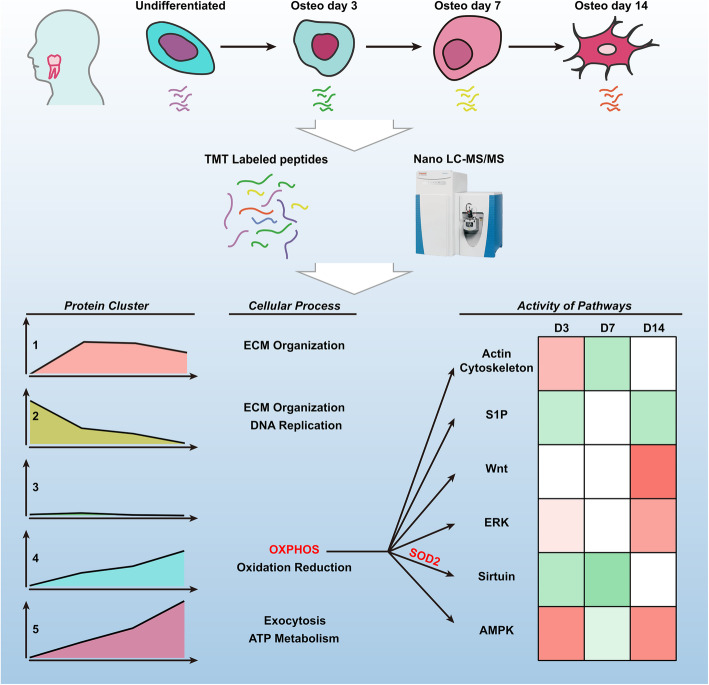

**Supplementary Information:**

The online version contains supplementary material available at 10.1186/s13287-020-02123-6.

## Introduction

Since they were first defined, human periodontal ligament stem cells (hPDLSCs) have been identified as the optimal seed cells for periodontal regeneration [1, 2]. They exhibit osteoblast-like characteristics and are capable of differentiating into cementoblasts or osteoblasts, showing great potential for the regeneration of functional periodontal tissues. Although efforts have been made in the past to promote the efficacy of osteogenesis of hPDLSCs, the clinical stem cell therapy effects are imprecise [3]. The exact molecular mechanism and signalling pathways that regulate differentiation potency remain to be deciphered.

Previous studies have conducted high-throughput techniques to reveal gene expression alterations in hPDLSCs at the transcriptional level. Regrettably, they focused only on regulatory non-coding RNAs rather than functionally translational genes, or focused only on a single time point (day 7 or 14 of osteogenesis duration) and the final phenotype [4–8]. Osteogenesis is a dynamic cellular differentiation process that consists of distinct developmental phases including proliferation and matrix synthesis/maturation/mineralization stages, indicating a phasic pattern of osteogenesis-related protein synthesis [9]. Zheng et al. investigated gene expression profiles altered continuously during the osteogenesis course of hPDLSCs and found some distinct expression patterns, such as constitutive downregulation of epigenetic regulators and dip patterns of osteoblast-associated genes [8]. Although protein abundances somehow scale with mRNA levels, this association was limitative to an extent and mRNA levels should not be interpreted as the final output of gene expression [10, 11]. This addresses the need to investigate the execution of cell differentiation with proteomic techniques, which reveal the regulatory mechanism at the translational level.

To date, only two reports have examined the proteomic profile during the osteogenic differentiation of hPDLSCs. As early as 2009, Xiao et al. compared the protein profile between osteogenic-induced and non-induced hPDLSCs using gel-based mass spectrometry [12]. They found that differentially expressed proteins were mainly related to the cytoskeleton, nuclear regulation, cell membrane binding, matrix synthesis, metabolic enzymes and signal transduction. Despite a new perspective on the osteogenic differentiation progress of hPDLSCs, the drawbacks of gel-based proteomic techniques, including low sensitivity, poor separation and resolution, undermine their application in accurate proteomic research [13]. Our team recently carried out research to explore the role of integrin alpha 5 in the osteogenic differentiation of hPDLSCs using a label-based technique [14]. This study involved protein profiling of late 14-day osteogenic differentiation to some extent, but it did not conduct an in-depth exploration of the dynamic protein expression pattern.

To address this question comprehensively, we conducted the first dynamic proteome analysis to reveal the temporal protein profile of hPDLSCs during the osteogenic differentiation. In the present study, we found that proteins involved in this process presented dynamic expression patterns and functioned distinctively. Oxidative phosphorylation (OXPHOS), the most significant pathway, was demonstrated to be continuously activated during the process. It was associated with several redox-sensitive signalling pathways (such as sirtuin and AMPK), which also showed altered activation/inhibition status. Furthermore, SOD2 might play a vital role in the osteogenesis of hPDLSCs.

## Methods

### Cell culture and osteogenic differentiation protocols

hPDLCs were obtained from three periodontally healthy donors (aged 20–25, molars or premolars) who requested tooth extraction for orthodontic treatment at the Department of Stomatology, Nanfang Hospital, Southern Medical University. This study was approved by the Ethics Committee of Nanfang Hospital, Southern Medical University (NFEC-2020-253), and informed consent was obtained from donors. The middle third of the periodontium was carefully scraped off with a surgical blade. After washing with PBS containing 100 U/mL penicillin/streptomycin, the periodontium was minced to approximately 1 mm^2^ per piece and scattered on the culture flask and maintained in low-glucose Dulbecco’s modified Eagle’s medium (Gibco, Invitrogen, NY, USA) with 10% foetal bovine serum (Gibco, Invitrogen, NY, USA) and 100 U/mL penicillin/streptomycin (Gibco, Invitrogen, NY, USA). After reaching 80% confluency, hPDLSCs were obtained utilizing the limiting dilution method, featuring clone formation capacity. Passages 4–6 of hPDLSCs were used in the subsequent experiments. We applied the chemically osteogenic differentiation protocol to induce osteogenesis of hPDLSCs. Briefly, hPDLSCs at a confluency of 80–90% were induced to differentiate using growth culture media supplemented with 10 mM beta-glycerophosphate (Sigma, St Louis, MO, USA), 50 μg/mL l-ascorbic acid-2-phosphate (WAKO, Osaka, Japan) and 100 nM dexamethasone (Sigma, St Louis, MO, USA). The osteogenic differentiation culture medium was changed every 3 days. Alkaline phosphatase (ALP) and alizarin red S (ARS) staining were performed to verify the establishment of the osteogenesis phenotype (details in supplementary materials).

### Cell sample preparation and Tandem Mass Tag (TMT) proteomics analysis

The detailed materials and methods are described in supplementary materials.

### Bioinformatic analysis

Proteins in each differentiated time point were compared to any other time point, and those with fold change > 1.2 or < 0.83 and *p* value < 0.05 determined by one-way ANOVA followed by post hoc Tukey HSD test were defined as differentially expressed proteins (DEPs). Correlations among different time points were calculated in Prism using Pearson correlation. To address the temporal dynamics of osteogenic differentiation of hPDLSCs, we subjected the DEPs to the k-means clustering algorithm assigning the optimal cluster number based on the reckon of sum of the squared errors. Functional enrichment analyses were subsequently performed for each cluster. Gene Ontology (GO) annotations were retrieved from the GO database (http://www.geneontology.org/), and hypergeometric test was used to find significantly enriched GO terms based on GO::TermFinder [15]. Pathway enrichment analysis was conducted in Metascape (http://metascape.org/gp/index.html) with the ontology source of Kyoto Encyclopedia of Genes and Genomes (KEGG) pathway using the *p* value set at 0.9 [16]. Ingenuity Pathway Analysis (IPA) was used to predict the activation/inhibition scores of canonical pathways [17]. The protein-protein interaction (PPI) network was built with the STRING online tool (https://string-db.org/) and visualized using Cytoscape. The minimum required score for PPI network construction was set to medium confidence (0.400) and the interaction derived from all provided sources including textmining, experiments, database, co-expression, neighbourhood, gene fusion and co-occurrence.

### qRT-PCR and western blotting analysis

Total RNA and proteins of osteogenic hPDLSC samples at days 0, 3, 7 and 14 were extracted and tested. Please see details in supplementary materials.

### Statistical analysis

GraphPad Prism was used to perform one-way ANOVA with post hoc Tukey HSD for comparisons among more than two groups. Significance was determined at *p* < 0.05. Data are presented as the mean ± SD. Band intensity in western blot images was quantified with ImageJ Software, and values are expressed as the means ± SD of at least three independent experiments. All experiments were performed at least in triplicate.

## Results

### Summary of TMT proteomics analysis

**Fig. 1:**
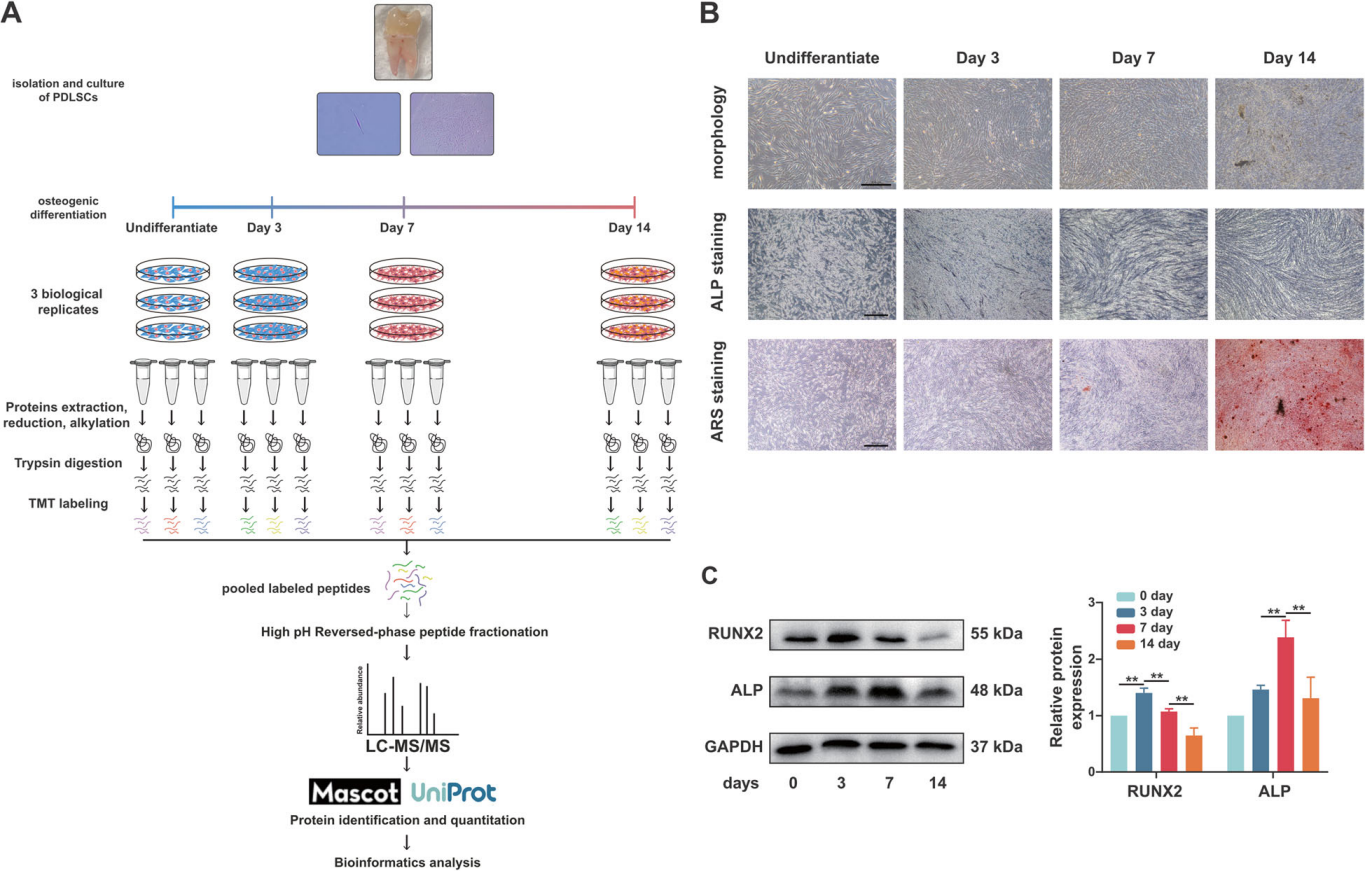
Schematic overview of the quantitative TMT proteomics workflow and validation of osteogenic differentiation of hPDLSCs. **a** Experimental overview of proteomic analysis of hPDLSCs during osteogenesis. hPDLSCs were isolated with limiting dilution methods and induced to undergo osteogenic differentiation. Protein lysates of cells induced for 0 (uninduced), 3, 7 and 14 days were collected. Following trypsin digestion of equal amounts of protein, peptides were labelled with 6-plex TMT reagents, fractionated by HPLC and analysed by LC-MS/MS. After protein identification and quantitation, bioinformatic analysis was performed. We performed three biological replicates of each time point. **b** Morphological characterizations of osteogenic-differentiated hPDLSCs. hPDLSCs induced for 0 (uninduced), 3, 7 and 14 days were imaged in bright field before ALP and ARS staining, respectively. Scale bar 500 μm. **c** Validation of osteogenesis-related proteins (RUNX2 and ALP) by western blotting. The data are represented as the means ± standard deviation (SD). **p < 0.01. All data were collected at least in triplicate

**Fig. 2:**
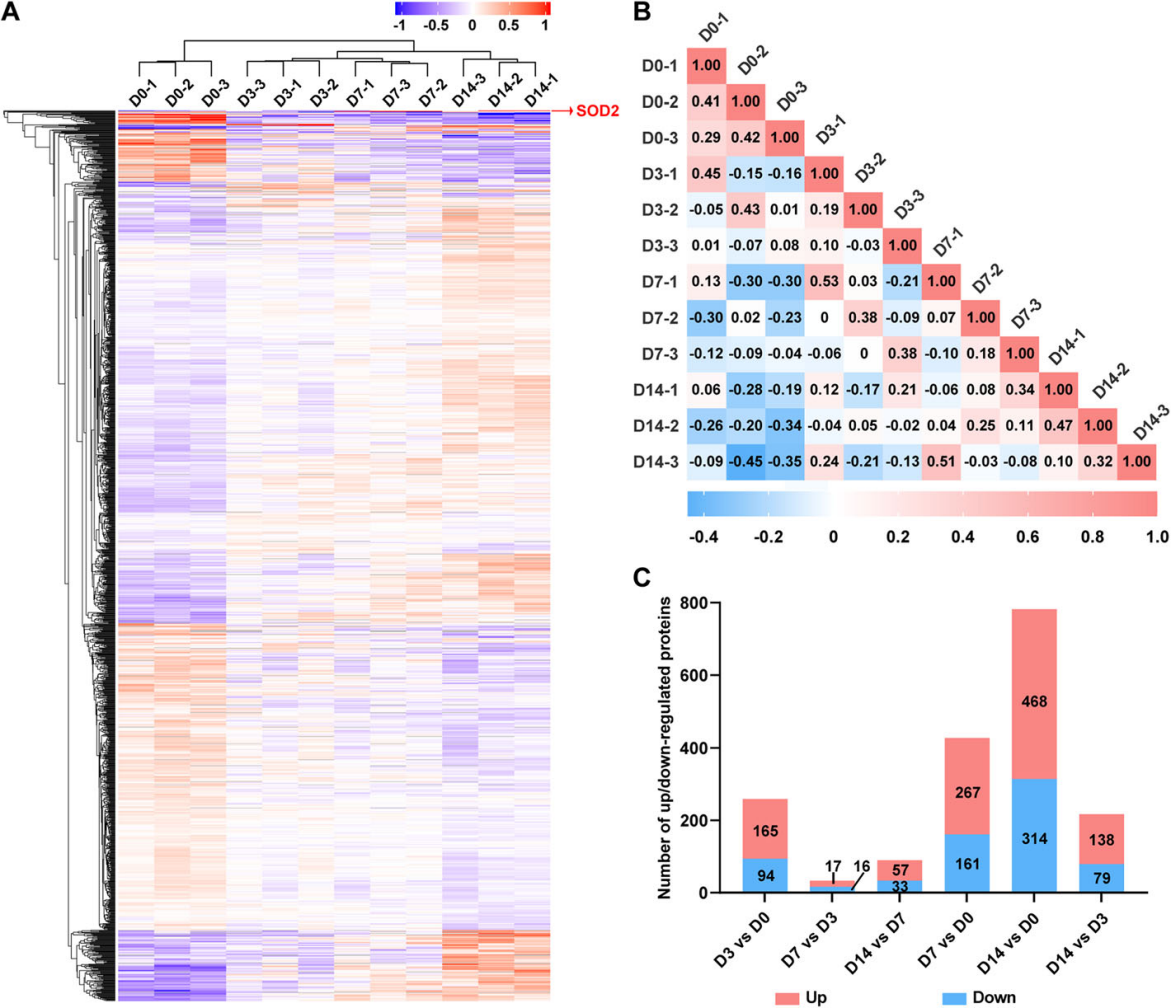
Characterization of proteomic profiles of hPDLSCs during osteogenic differentiation. **a** Overall profiles of proteins at four time points during osteogenesis of PDLSCs. **b** Pairwise correlation analyses of samples. The results are presented in the form of correlation matrixes. One matrix represents the overall protein expression of one sample. The colour of the square indicates the magnitude of the correlation, and the colour bar refers to the correlation coefficient. The number inside the square indicates the exact correlation coefficient between any two time points. **c** The number of DEPs across time points. The red sections of the bar indicate upregulated proteins, and the blue sections of the bar indicate downregulated proteins

To describe the proteome dynamics during osteogenic differentiation of hPDLSCs, we used the classical induction protocol to induce osteogenic-differentiated hPDLSCs (Fig. 1a). During the 14-day differentiation, the ALP activity continuously increased with a rapid increase at day 7, and the mineralized nodules were obvious at day 14 in the bright field as well as after ARS staining (Fig. 1b). Consistent with the morphological changes, osteogenesis-related proteins (RUNX2 and ALP) showed dynamic changes at the protein level (Fig. 1c). We then conducted TMT proteome analyses at days 0, 3, 7 and 14. A total of 7012 proteins with at least one unique peptide sequence and 1% false discovery rate confidence were identified, and the protein expression profiles at 4 time points are presented in Fig. 2a. We performed subsequent analysis on a set of 5806 proteins quantified across all time points with at least two replicates (variations of quantification data among biological samples were shown in Fig. S1). As shown in Fig. 2b, the correlation coefficients by Pearson correlation analysis were low between different time points, indicating that the protein profile among different osteogenesis phases was significantly distinct.

A total of 1024 DEPs were identified, and the number of DEPs across all time points is illustrated in Fig. 2c (details in Table S1). Proteins differed the most between the undifferentiated stage (D0) and terminal differentiated stage (D14). Among the 1024 DEPs, proteins associated with osteogenesis are listed in Table 1, including ALP and collagen proteins (COL1A1, COL1A2, COL3A1 and COL6A1) as well as some transduction signalling molecules (PDGFRA and TGFBR1). For FBN1, we have previously validated its involvement in osteogenic/odontongenic differentiation of dental pulp stem cells [47].

**Table 1:** Osteogenesis-related proteins with differential expressions in the TMT proteomics

Uniprot ID	Protein name	Gene name	Cluster^**a**^	Reference
Q12802	A-kinase anchor protein 13	AKAP13	1	[18]
P07355	Annexin A2	ANXA2	1	[19]
P35556	Fibrillin-2	FBN2	1	[20]
Q04771	Activin receptor type-1	ACVR1	2	[21]
P02765	Alpha-2-HS-glycoprotein	AHSG	2	[22]
O00622	CCN family member 1	CCN1	2	[23]
P02452	Collagen alpha-1(I) chain	COL1A1	2	[24]
P08123	Collagen alpha-2(I) chain	COL1A2	2	[24]
P02461	Collagen alpha-1(III) chain	COL3A1	2	[25]
Q96CG8	Collagen triple helix repeat-containing protein 1	CTHRC1	2	[26]
O75369	Filamin-B	FLNB	2	[27]
P19883	Follistatin	FST	2	[28]
P17302	Gap junction alpha-1 protein	GJA1	2	[29]
P35052	Glypican-1	GPC1	2	[30]
O60565	Gremlin-1	GREM1	2	[31]
P50281	Matrix metalloproteinase-14	MMP14	2	[32]
Q32P28	Prolyl 3-hydroxylase 1	P3H1	2	[33]
Q92791	Endoplasmic reticulum protein SC65	P3H4	2	[34]
P16234	Platelet-derived growth factor receptor alpha	PDGFRA	2	[35]
P50454	Serpin H1	SERPINH1	2	[36]
P36897	TGF-beta receptor type-1	TGFBR1	2	[37]
P37275	Zinc finger E-box-binding homeobox 1	ZEB1	3	[38]
Q03135	Caveolin-1	CAV1	4	[39]
Q9UP38	Frizzled-1	FZD1	4	[40]
P17936	Insulin-like growth factor-binding protein 3	IGFBP3	4	[41]
A1X283	SH3 and PX domain-containing protein 2B	SH3PXD2B	4	[42]
P05186	Alkaline phosphatase, tissue-nonspecific isozyme	ALPL	5	Not list out
P12109	Collagen alpha-1(VI) chain	COL6A1	5	[43]
P35555	Fibrillin-1	FBN1	5	[44]
Q13491	Neuronal membrane glycoprotein M6-b	GPM6B	5	[45]
P52926	High mobility group protein HMGI-C	HMGA2	5	[46]

^a^Clusters from k-means clustering of Fig. 3

### Dynamic expression and functional characterization of DEPs

**Fig. 3:**
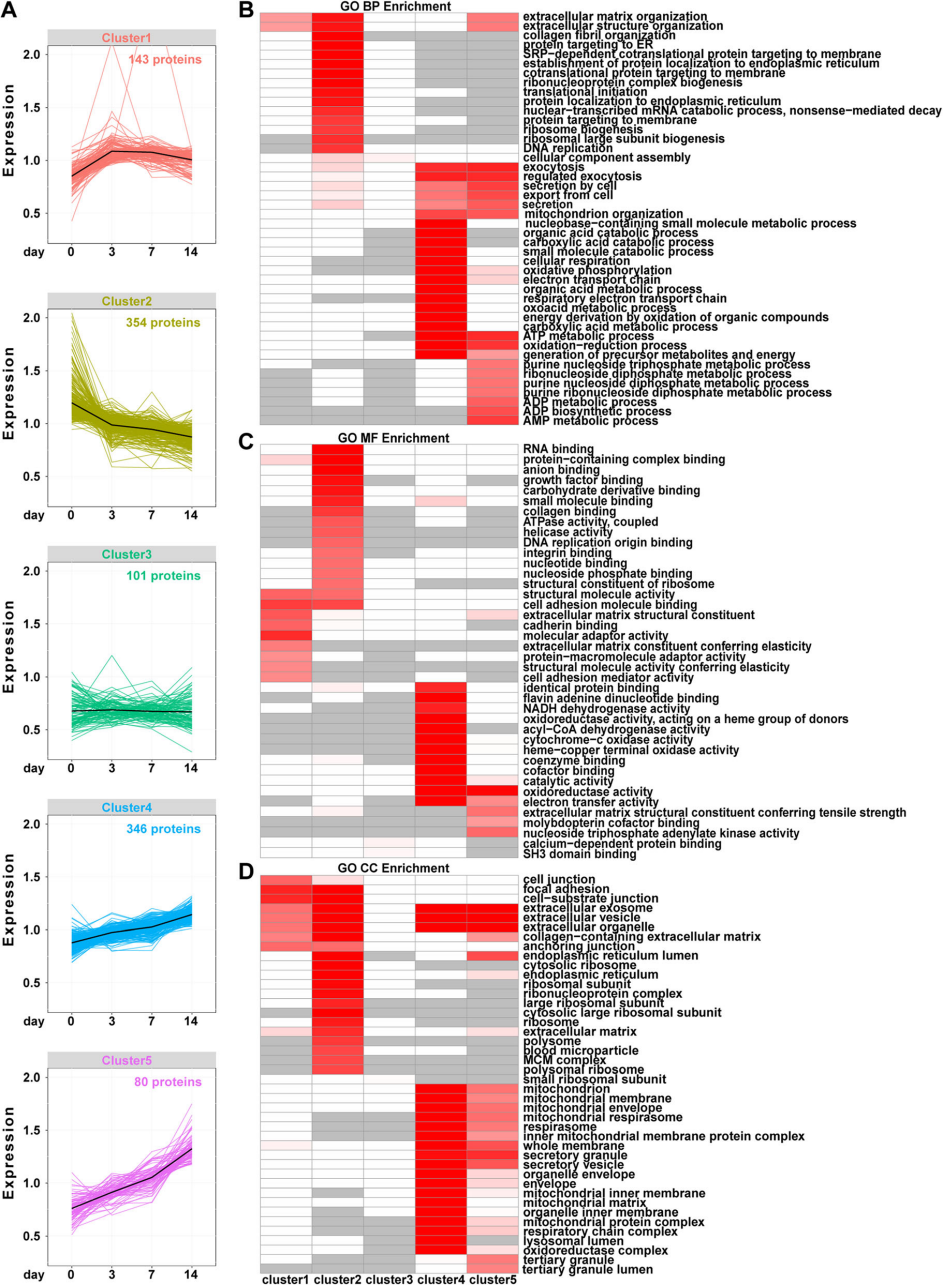
Functional annotation clustering of the differentially expressed proteins. **a** Five clusters of the 1024 differentially expressed proteins. Different clusters are illustrated in different colours. **b** Representative biological processes of five clusters. BP, biological process. **c** Representative molecular functions of five clusters. MF, molecular function. **d** Representative cellular components of five clusters. CC, cellular component. The colours in **b**, **c** and **d** represent the enrichment results. Darker red indicates a smaller *p* value while lighter red indicates larger *p* value. White stands for items with nonsignificant *p* values. Grey indicates that this item was not enriched in this cluster. The details are provided in Supplementary Table 2

To better monitor the temporal changes in protein expression, we subjected the 1024 DEPs to the k-means clustering algorithm. The optimal number of clusters was 5 (data not shown). Therefore, the quantitative temporal profiles partitioned into 5 clusters (Fig. 3a). The largest cluster was cluster 2, with 354 proteins whose expression continuously declined from day 0. The second largest cluster was cluster 4, with 346 proteins whose expression gradually increased during the whole differentiation course. Meanwhile, the expression of 80 proteins in cluster 5 changed similarly to that of proteins in cluster 4 but showed a more rapid increase tendency. Cluster 1 was composed of 143 proteins that peaked at day 3 and showed a small decrease or remained thereafter. Cluster 3 consisted of 101 proteins whose expression remained at the similarly level during the course.

Considering that proteins with different expression patterns may perform different functions, we tested the respective enrichment of GO terms. Generally, the GO enrichment results indicated that proteins with different tendencies show different functions. The results of GO enrichment are illustrated in the form of heatmaps (Fig. 3b–d, details in Table S2). Cluster 3, with proteins that changed little during the differentiation course, was not significantly enriched in any functional categories. Meanwhile, clusters 4 and 5 seemed to share similar terms to some extent.

Cluster 2, with proteins that continuously declined upon initiation of osteogenic differentiation, was enriched in DNA replication categories (Fig. 3b). This biological process (BP) term consisted of proteins involved in cell cycle progression, such as the cyclin-dependent kinase CDK9 and the minichromosome maintenance (MCM) protein family, which occupy a central role in DNA synthesis [48]. Another commonly used proliferative marker PCNA and embryonic stem cell marker SSRP1 [49] were also found in cluster 2. Cluster 1, where proteins increased to peak at day 3 and then declined slightly or maintained the expression level, was enriched in the extracellular matrix (ECM) and structure organization. The proteins involved included the osteogenic-related proteins annexin A2 (ANXA2), fibrillin-2 (FBN2) and fibulins (FBLN1 and FBLN5). For cluster 4, mitochondrial function, cellular respiratory process, OXPHOS, secretion function and catabolic process were obviously enriched. Meanwhile, the BP function of proteins from cluster 5 overlapped somewhat with that of cluster 4, but cluster 5 may also participate in cellular energy metabolism. Interestingly, the functions of ECM organization and structure were enriched in clusters 1, 2 and 5, representing different alteration tendencies. COL6A1, COL6A2, COL6A3 and COL18A1 belonged to cluster 5, while COL8A1 and COL11A1 were from cluster 1. COL1A1, COL1A2, COL3A1, COL5A1 and COL14A1 were from cluster 2.

When referring to molecular function GO analysis (Fig. 3c), functions distinguished more among different clusters. Cluster 1 describes the early osteogenesis phase and is involved in cell adhesion, molecular adaptors, structural molecules and ECM-related constituents. Cluster 2 described proteins that bind to diverse macromolecules, including RNA, DNA, growth factor and collagen. Clusters 4 and 5 both described proteins involved in the activity of oxidoreductase, catalytic and electron transfer. Cluster 4 also represented proteins associated with the activity of mitochondrial function, while proteins related to ECM structural constituent and nucleoside triphosphate adenylate kinase activity were for cluster 5.

In Cellular Component GO analysis (Fig. 3d), proteins from clusters 1 and 2 shared the same categories (extracellular organelle and cell junction/adhesion) to some degree, despite they representing different alteration tendencies. This was also the case between clusters 4 and 5, where proteins involved in mitochondrial constituents and secretory transporters presented an upregulation during the course of osteogenesis of hPDLSCs either in a moderate or rapid trend.

In summary, GO enrichment demonstrated that proteins associated with ECM organization/interaction, secretory function and mitochondrial activity played functions in a complex regulatory manner during osteogenesis of hPDLSCs.

### OXPHOS was the most enriched pathway in KEGG analysis

**Fig. 4:**
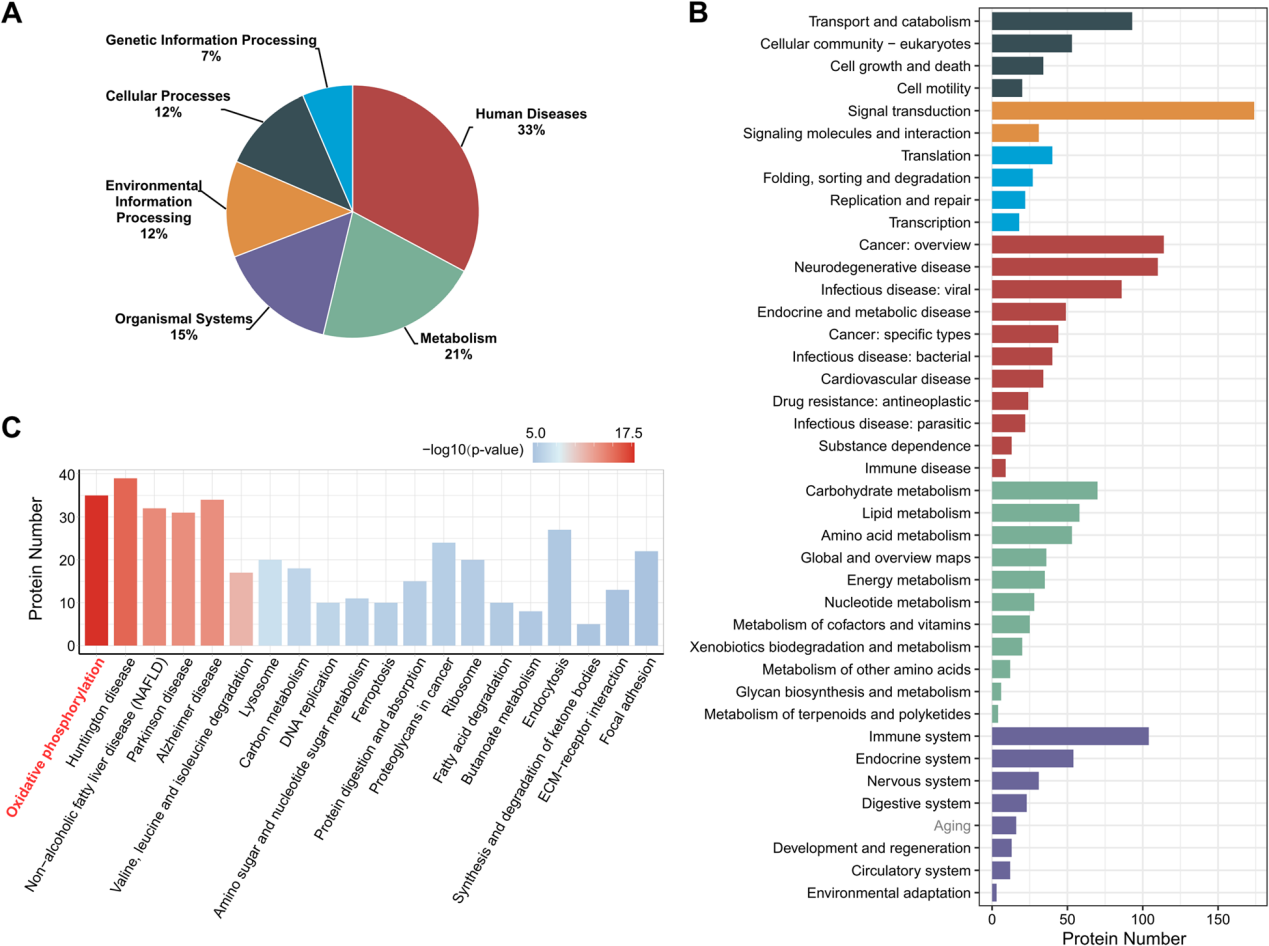
Pathway annotation of differentially expressed proteins by the KEGG database. **a** The classification of differentially expressed proteins by KEGG database annotation at level 1. **b** The classification of differentially expressed proteins by KEGG database annotation at level 2; colour legends were the same as in **a**. **c** KEGG pathway enrichment analysis of differentially expressed proteins

A total of 1022 out of 1024 DEPs were mapped to the KEGG database using Metascape (http://metascape.org/gp/index.html), and their functional annotations were classified (Fig. 4a, b). The second largest pathway classification was “Metabolism”, annotated to 50 metabolic pathways occupying 21% (347) of 1022 DEPs. As shown in Fig. 4b, most of the DEPs (174) were clustered in the “Signal transduction” classification at level 2, annotated to 20 specific signalling pathways including osteogenesis-related pathways such as PI3K-Akt, HIF-1, mTOR, FoxO, TGF-β, AMPK, MAPK and Wnt signalling pathways (detailed enrichment results in Table S3). The top enriched pathways included metabolism-related pathways, biogenesis of proteins and amino acids, DNA replication, organelles, endocytosis and ECM interactions (Fig. 4c). It is worth noting that “oxidative phosphorylation” term was the most significant pathway.

### Dynamic activation of redox-sensitive signalling pathways revealed by IPA

Apart from the pathways in which the DEPs participated, we also examined whether these pathways were suppressed or activated during the course. Here, we used IPA to identify activation or inhibition of the functional pathways. By comparing one time point with the previous one, we observed that various pathways showed altered activated/inhibited status. A total of 75 Ingenuity Canonical Pathways had valid data at least two time points (Table S4).

**Fig. 5:**
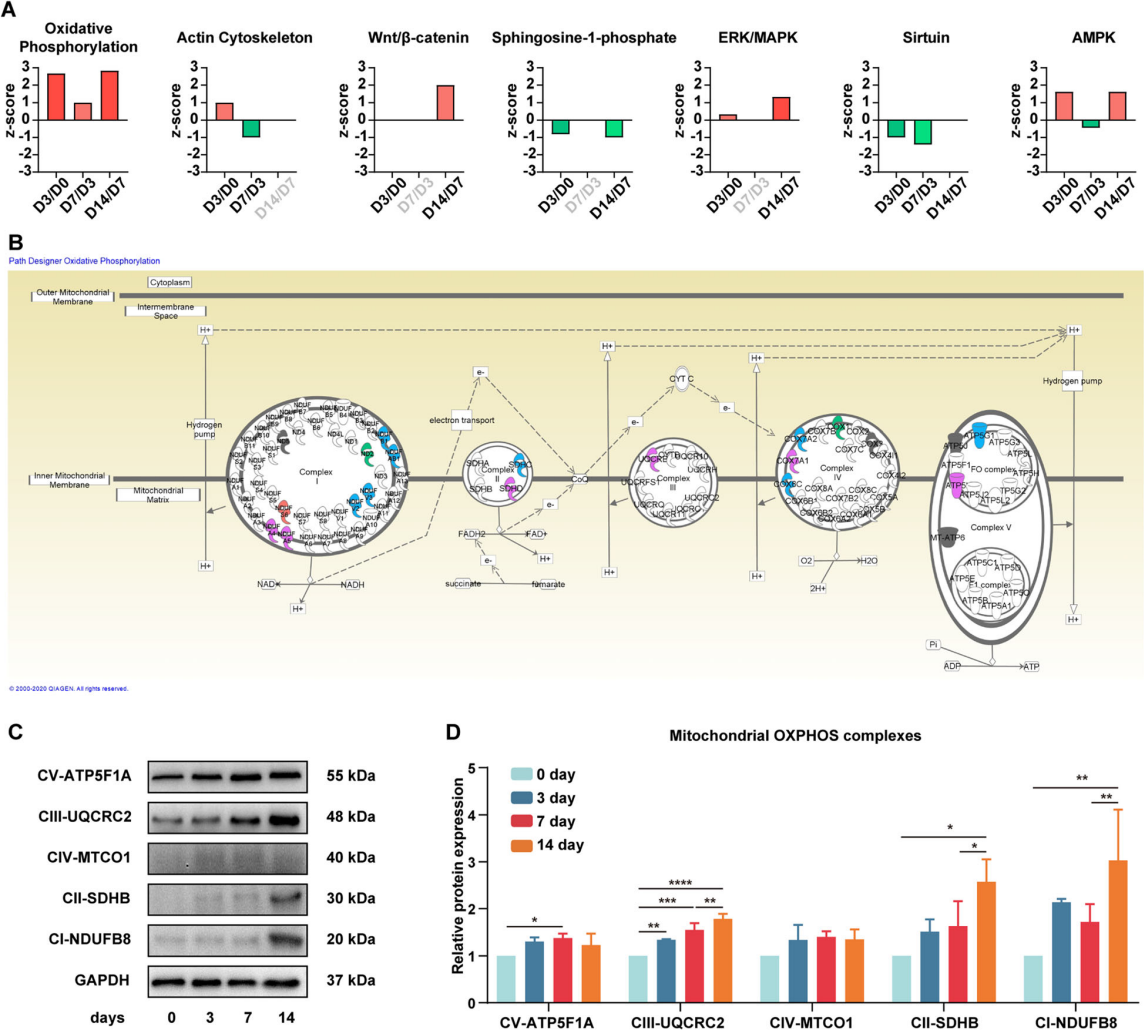
Activation prediction of the oxidative phosphorylation (OXPHOS) pathway and redox-sensitive signalling pathways. **a** Activation status of the OXPHOS pathway and redox-sensitive signalling pathways. A positive *z*-score represented the predicted activation and is illustrated in red; a negative *z*-score represented the predicted inhibition and is illustrated in green. The lighter the colour is, the larger the |*z*-score| is. The time point labelled in light grey means no valid prediction. **b** Map of the “oxidative phosphorylation” canonical pathway in the Ingenuity Pathway Analysis database. Proteins labelled in red, green, blue and pink means proteins from clusters 1, 3, 4 and 5 of differentially expressed proteins, respectively. Grey indicates the proteins are not differentially expressed. **c** Validation of representative proteins from five OXPHOS complexes by western blotting. GAPDH was used as a loading control. **d** The quantitative results of western blotting from Fig. 5c, data are represented as the means ± standard deviation (SD). *p < 0.05; **p < 0.01; ***p < 0.001; ****p < 0.0001. All data were collected at least in triplicate

Undoubtedly, “oxidative phosphorylation” canonical pathway was also enriched in the IPA database with continuous activation in all three stages (Fig. 5a, b). The upregulations of 5 mitochondrial complexes were verified with western blotting (Fig. 5c). Noticeably, UQCRC2 (from complex III subunit), SDHB (from complex II subunit) and NDUFB8 (from complex I subunit) showed a remarkable increase during osteogenesis. Combined with the apparent effect of mitochondria revealed by GO analysis, we reasoned into the probable vital role of OXPHOS inside the mitochondria during the osteogenic differentiation of hPDLSCs. Increased mitochondrial activity and high levels of OXPHOS are inevitably accompanied by reactive oxygen species (ROS) by-products, which are thought to be primarily generated in mitochondria [50]. ROS can regulate a diverse array of physiological processes via oxidation of signalling molecules [51, 52]. In addition, molecular signal transduction plays a crucial role in the osteogenic differentiation of stem cells [53]. Thus, we further examined the time course changes in the activation profiles of the ROS-regulated signalling pathway, namely, redox-sensitive signalling pathways in IPA.

Based on a literature search, 6 redox-sensitive signalling pathways were closely related to osteogenesis. These pathways presented a dynamic activity profile (Fig. 5a and the detailed map in Fig. S2, S3, S4, S5, S6, S7). Wnt signalling was predicted to be non-activated at day 3 but activated at day 14. Sphingosine-1-phosphate (S1P) and sirtuin signalling pathways were predicted to be inhibited during the course, where the S1P signalling pathway was inhibited at days 3 and 14, while the sirtuin signalling pathway was suppressed at the early and mid-term course and showed no inhibition at the terminal stage. The ERK/MAPK signalling pathway represented activation status at days 3 and 14; however, the status of day 7 was not indicated in our data. Actin cytoskeleton signalling, a cytoplasmic structure-associated pathway, was predicted to be activated at the early osteogenesis stage but inhibited at the mid-term. Similarly, such dynamic changes could also be observed in the AMPK signalling pathway with activation at days 3 and 14 but inhibition at day 7.

From the above, redox-sensitive signalling transduction pathways might play roles in osteogenesis of hPDLSCs in a temporal manner, in line with the dynamic change in this cellular process.

### The interactions between OXPHOS and redox-sensitive signalling pathways

**Fig. 6:**
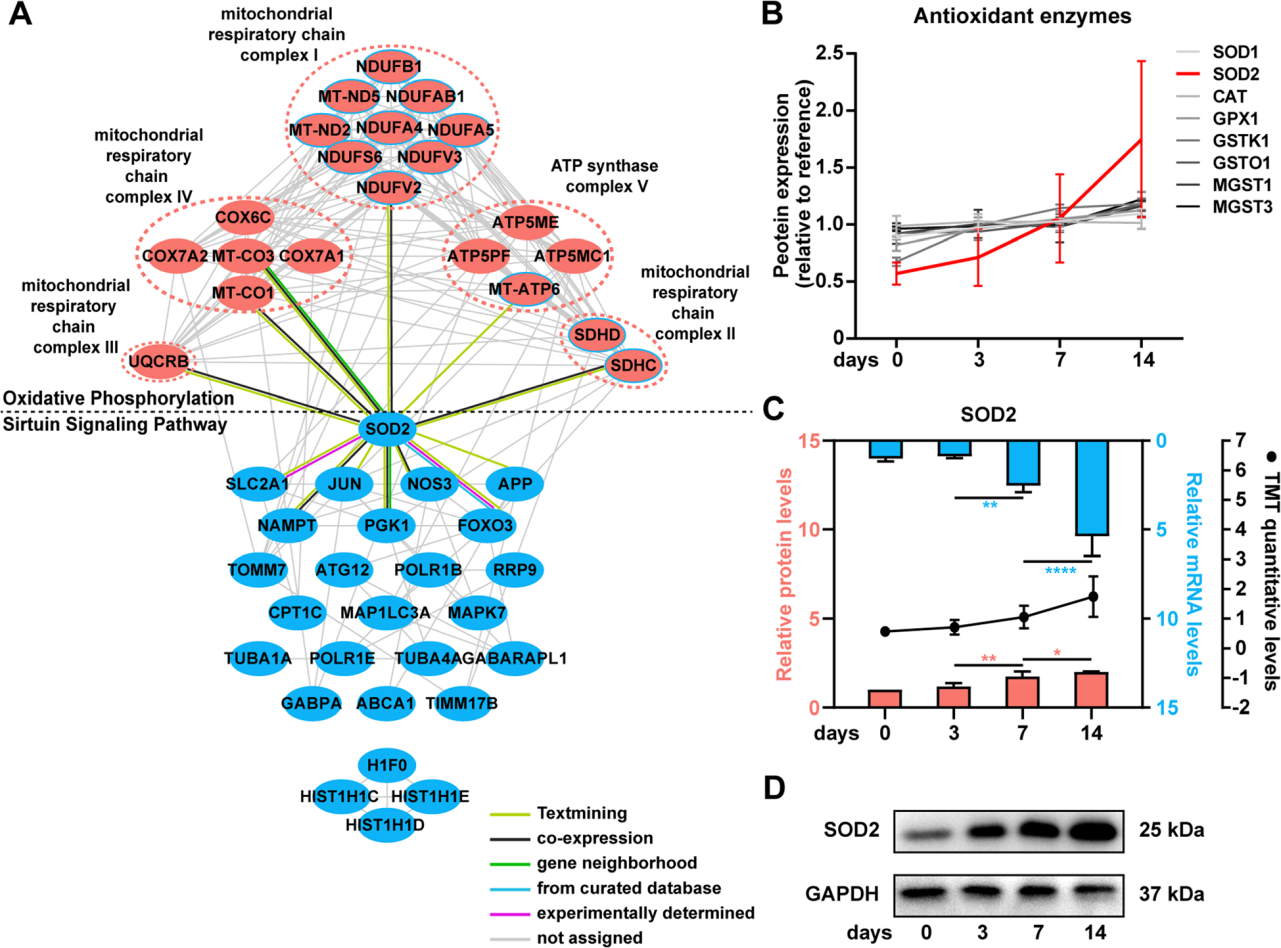
SOD2 acted as the hub protein to associate oxidative phosphorylation (OXPHOS) and sirtuin signalling pathway. **a** The protein-protein interaction network of OXPHOS and the sirtuin signalling pathway. Protein circled with blue in OXPHOS indicated that they were also parts of the sirtuin signalling pathway. **b** The protein expression of all 8 antioxidant enzymes detected in the proteome data and SOD2 showed the most remarkable upregulation. **c** The combination chart showing the expression of SOD2 detected with qRT-PCR, western blotting and TMT methods. The upper columns represent the mRNA expression levels, the bottom columns represent the grey value of the western blotting and the line chart represents the TMT quantitative expression. The data are represented as the means ± standard deviation (SD). *p < 0.05; **p < 0.01; ****p < 0.0001. All data were collected at least in triplicate. **d** Representative bands of SOD2 protein. GAPDH was used as a loading control

To reveal the potential correlations between OXPHOS and redox-sensitive signalling pathways, a PPI network was utilized. As shown in Fig. S8, all six pathways were related to OXPHOS to varying degrees: 21 edges for sirtuin, 15 edges for AMPK, 7 edges for Actin cytoskeleton, 3 edges for S1P and 2 edges for both ERK/MAPK and Wnt/β-catenin signalling. The sirtuin signalling pathway not only correlated most but also shared the most components with OXPHOS. Illustrated with a detailed network (Fig. 6a), we found that SOD2, a main antioxidant, showed a high connection degree of 12 edges in the network. Additionally, among all eight antioxidants detected in our data, SOD2 showed the most remarkable upregulation within the course (Fig. 6b). Hence, we validated SOD2 mRNA and protein expression and found an upregulation starting at day 3 and a remarkable increase at day 14, consistent with the TMT data (Fig. 6c, d).

## Discussion

Osteogenic differentiation is a progressive physiological process, and related genes/proteins are expressed in a temporal and coordinating manner, which cannot be fully evaluated by limited morphological, cytochemical and biochemical testing. The present study was motivated by questions on the temporal dynamics of protein profiling during the osteogenic differentiation of hPDLSCs. Using label-based quantitative proteomics, which allowed the simultaneous profiling of approximately 7000 proteins of diverse time points, we first presented a large-scale dynamic proteomic profiling of this process. With clustering and functional annotation, we characterized the proteins from different expression patterns with distinct functions. Subsequently, we mapped activation profiles of canonical signalling pathways, which revealed the interactions between OXPHOS and redox-sensitive signalling pathways. Furthermore, SOD2, a continuously upregulated antioxidant enzyme, might coordinate the sirtuin signalling pathway and OXPHOS during the osteogenic differentiation process.

Clustering the proteins along with functional annotation helped us to identify the distinct functions of different protein classes. The quantitative temporal profiles were partitioned into 5 clusters. In the bone tissue lifespan, ECM is not a static but dynamic microenvironment with innate complexities, making it important in homeostasis and regeneration spatially and temporally [54, 55]. In our study, different types of ECM showed different expression patterns. Surprisingly, COL1A1 and COL1A2, along with other type I distribution-associated collagens, including COL3A1, COL5A1 and COL14A1 [56], continuously decreased. This was inconsistent with many other studies reporting increased *COL1* mRNA expression during osteogenesis of MSCs. Nevertheless, a negative relationship was found between collagen mRNA levels and cell layer collagen protein accumulation in differentiated pre-osteoblasts [57]. Collagen synthesis occurs early in the life of these cultures, whereas insoluble collagen deposition occurs later accompanied by a diminished rate of collagen synthesis. In the presence of ascorbic acid, which facilitates osteogenic differentiation by increasing collagen type 1 secretion [58], collagen type I protein was secreted and assembled into extracellular fibrils in a time-dependent manner while the intracellular collagen type I protein decreased [59]. Along with the knowledge of the high intrinsic expression levels of *COL1* mRNA in hPDLSCs [60–62], we suspected that the intrinsic intracellular collagen type I protein might be adequate for osteogenic differentiation and secretory function rather than protein synthesis playing a major role in this cellular differentiation process. However, further studies concerning the association between the intrinsic and secreted proteins during this process are needed.

COL6s, in contrast to COL1s, were increased throughout the course. In human osteoblast SaM-1 cells, the decline of *COL6A1* at the mRNA and protein levels decreased *COL1A1* mRNA in the early phase of mineralization [63]. Another ECM FBLN1 was reported to be indispensable for the good organization of collagen fibre bundles but did not affect its expression in mouse PDLs [64]. These results suggested the potential association between different types of ECM expressionally or functionally. The altered ECM protein pattern in our study coincided with the notion that the ECM is constantly undergoing remodelling. Nonetheless, to investigate the exact roles of ECM in the osteogenesis of hPDLSCs, methods for the detection of secretory proteins and ECM depositions should be conducted, not merely intracellular protein quantifications [65].

Compared to our results in PDLSCs, the ECM of MSC from the bone marrow presented an upregulation during osteogenesis, despite the detection at mRNA levels [9]. It is a pity that so far, no study has investigated the dynamic protein expression patterns of BMSC during osteogenic differentiation. Comprehensive investigations should be carried out to answer the question whether these dynamic ECM expression patterns in MSCs were distinctive among origins.

Commonly, the mitochondria maintained a relatively low activity level in undifferentiated MSCs while activated in differentiated cells [66]. Upon osteogenic induction of hBMSCs, mitochondrial biogenesis was intensified where the copy number of mitochondrial DNA, protein subunits of the respiratory enzymes and intracellular ATP content were increased [67]. In our study, mitochondrial function was also activated, suggested by the upregulation of proteins functioned in mitochondrial activity and cellular energy-producing catabolic processes. Single-stranded DNA-binding protein, which is implicated in replication and maintenance of mitochondrial DNA [68], belonged to cluster 4 and continuously increased. The respiratory electron transport chain (ETC), located on the mitochondrial inner membrane, is composed of complexes I–IV of the respiratory chain and complex V, an ATP synthase, driving ATP synthesis [69]. The ETC-related proteins all presented an increasing tendency in our study, indicating the high activity of the cellular respiration and the energy production. The well-known function of mitochondria is to provide energy for cell life activities through OXPHOS, which occurs in the mitochondrial cristae [70]. This was also detected in our study, where the proteins from GO terms of OXPHOS, metabolic process and inner mitochondrial membrane complex tended to be upregulated.

The function of OXPHOS was further clarified in the KEGG pathway and IPA analyses. The high level of OXPHOS along with alterations in the distribution, number, mass and morphology of mitochondria are important characteristics during the osteogenic differentiation of MSCs [71], which corresponds with the increased protein expressions of OXPHOS complexes in western blotting validation. Commonly, stimulation of osteogenic differentiation seems to suppress adipogenic differentiation and this biological basis is increasingly implicated by the tight regulation by ROS, the by-products of OXPHOS. A study has revealed that the intracellular levels of ROS were dramatically reduced during the first 2 days of osteogenic induction but as osteogenic differentiation continued, gradual rebound ROS was noted and higher than that of undifferentiated hMSCs at the terminal stage of day 28 [67]. However, in another study, the ROS level in BMSCs was significantly continuously increased during osteogenesis [72]. Contradictories exist and the ROS level cannot be predicted by the activity of OXHPOS; hence, how the ROS level is altered during the osteogenic differentiation of hPDLSCs is urgently needed in future studies.

An increasing number of studies have paid more attention to the potential involvement of ROS in the signalling pathways [73, 74], i.e. redox-sensitive signalling pathways. Using IPA, we noticed that several redox-sensitive signalling pathways presented dynamic activities during the osteogenic differentiation of hPDLSCs as well as the connections with OXPHOS. Among these, the sirtuin signalling pathway was associated the most, in which SOD2 was one of the hub proteins. From the PPI, we noticed that numerous mitochondrial proteins belonged to both the pathways, indicating the intrinsic relationship between them. Sirtuins belong to the class III histone deacetylase (Hdac) family, named for their homology of the yeast protein silent information regulator (Sir)2 [75]. Emerging studies have discussed the functions of different sirtuins in the field of osteogenesis during the last decade. SIRT1 mediated the enhancement of osteogenic differentiation by exogenous antioxidants [76, 77]. Upregulation of SIRT3 was found during the antioxidation by melatonin in osteogenesis along with strengthening the activity of SOD2 [78]. Similarly, SIRT6 was also positively related to the osteogenic phenotype, in which downregulation of SIRT6 could inhibit osteogenesis of murine BMSCs, while overexpression could reverse it [79]. However, downregulation of SIRT7 improved the osteogenic differentiation of hBMSCs by activating β-catenin [80]. In our study, inhibited activity was found on day 3 and day 7, while non-activation was predicted in the terminal state. The function of sirtuins in osteogenesis and bone metabolism has just come into pictures, and the complexity and dual effects of sirtuins make it worthy of in-depth study. Although the sirtuin proteins did not show altered expression in our data, the downstream targets and other proteins participating in the sirtuin signalling pathway presented dynamic expression. FOXO3, a downstream protein of SIRT1, presented an upregulation at an early stage but maintained its expression after that in our study. A study found that in addition to the increasing expression of FOXO3a, overexpressed SIRT1 also deacetylated FOXO3a, thus leading to the restoration of osteogenesis [81], indicating that there might be a modulation of FOXO3 at the transcriptional and posttranscriptional levels in our study. SOD2 was the prominent antioxidant enzyme during hPDLSC osteogenesis and was also identified as an upregulation pattern in the osteogenesis of stem cells of other types [67]. The levels of antioxidants are crucial for the correct lineage differentiation and osteogenesis potency of MSCs by modulating the antioxidative ability, redox-sensitive signalling pathway and mitochondrial function/activity [50]. *SOD2* was reported to be transcriptionally regulated by FOXO3 in the presence of dysregulated ROS [82], and there was a positive association between FOXO3 and SOD2 at the protein level via a Sirt1-mediated pathway during osteoblastic bone formation [81]. The specific role of SOD2 and its exact relationship with FOXO3a in osteogenesis-related redox-sensitive pathways are worthy of further investigation.

Two signalling pathways showed dual dynamic activity in our study, namely, actin cytoskeleton signalling and AMPK signalling. The impaired migration ability of MSCs was associated with excessive cellular ROS which led to overpolymerization of the F-actin cytoskeleton [83]. In the field of neuronal development and trafficking, the regulation of the cytoskeleton dynamics by differential ROS levels has been investigated [84]. The dynamic actin cytoskeleton activity might be ROS-involved during the osteogenic differentiation of hPDLSCs. The AMPK signalling pathway, a widely studied pathway in cellular redox regulation, is activated by the increase in cellular ROS and conversely, it can also decrease ROS levels by increasing transcriptional regulation of antioxidant defences, including catalase and SOD2 via activation of PGC-1α and FOXO3 [85]. A previous study [86] has demonstrated that AMPK activation was required at early differentiation but downregulation of AMPK at the mid-late period was also necessary. This was consistent with our findings despite the different durations of differentiation. Activation of AMPK at an early stage augments autophagy and leads to the initiation of differentiation, while accumulation of autophagosome components could impair differentiation, which could be downregulated by AMPK blockage. This suggests the necessity of dynamic signalling pathways as for their potential dual effects in different differentiated stages.

Other redox-associated signalling pathways, including Wnt/β-catenin signalling [87], S1P signalling [88] and the ERK/MAPK signalling pathway [89], also presented dynamic activity in our study and might regulate osteogenic differentiation in a temporal-dependent manner, where the activation status of the ERK/MAPK signalling pathway has been verified in a previous study [90].

At last, from our original data with respect to correlations among all samples, the intra-group correlation was not as high as expected (Fig. 2b). This might be attributed to the intrinsically donor-to-donor and intra-population heterogeneity, which often weakens the reproduction of experimental results in a certain degree [91, 92]. This suggested that we might need to expand the sample size in the future for further extrapolation of our conclusions. The sample size of the current study should be sufficient to draw a preliminary conclusion; however, we should take the sample size into account when it comes to primary mesenchymal stem cells.

## Conclusion

Collectively, with the use of a large-scale quantitative proteomic technique, we first described the dynamic protein expression pattern during osteogenic differentiation of hPDLSCs. Biological functions differed among various clusters of proteins with different alteration tendencies. Pathway enrichment along with the activation prediction suggested that OXPHOS might play a vital role in the process, and the sirtuin signalling pathway was closely associated with OXPHOS. Furthermore, SOD2, associating the aforementioned two pathways, was confirmed to be continuously upregulated, representing the activation of the antioxidant system in the osteogenesis of hPDLSCs. Our comprehensive proteomics analysis demonstrated a dynamic regulatory mechanism during the osteogenic differentiation of hPDLSCs and might provide a new perspective for research on periodontal regeneration.

## Supplementary Information

**Additional file 1: Figure S1.** Boxplots of normalized protein ratio of all 12 samples.

**Additional file 2: Figure S2.** Map of the “Sirtuin signalling pathway” canonical pathway in the Ingenuity Pathway Analysis database. Proteins labelled in red, yellow, green, blue and pink are proteins from clusters 1, 2, 3, 4 and 5 of differentially expressed proteins, respectively. Grey indicates proteins that are not differentially expressed.

**Additional file 3: Figure S3.** Map of the “Actin Cytoskeleton signalling pathway” canonical pathway in the Ingenuity Pathway Analysis database. Proteins labelled in red, yellow, green, blue and pink are proteins from clusters 1, 2, 3, 4 and 5 of differentially expressed proteins, respectively. Grey indicates proteins that are not differentially expressed.

**Additional file 4: Figure S4.** Map of the “AMPK signalling pathway” canonical pathway in the Ingenuity Pathway Analysis database. Proteins labelled in red, yellow, green, blue and pink are proteins from clusters 1, 2, 3, 4 and 5 of differentially expressed proteins, respectively. Color grey referrer to the proteins not differentially expressed.

**Additional file 5: Figure S5.** Map of “ERK/MAPK signalling pathway” canonical pathway in the Ingenuity Pathway Analysis database. Proteins labelled in red, yellow, green, blue and pink are proteins from clusters 1, 2, 3, 4 and 5 of differentially expressed proteins, respectively. Grey indicates proteins that are not differentially expressed.

**Additional file 6: Figure S6.** Map of the “Sphingosine-1-phosphate signalling pathway” canonical pathway in the Ingenuity Pathway Analysis database. Proteins labelled in red, yellow, green, blue and pink are proteins from clusters 1, 2, 3, 4 and 5 of differentially expressed proteins, respectively. Grey indicates proteins that are not differentially expressed.

**Additional file 7: Figure S7.** Map of the “Wnt/β-catenin signalling pathway” canonical pathway in the Ingenuity Pathway Analysis database. Proteins labelled in red, yellow, green, blue and pink are proteins from clusters 1, 2, 3, 4 and 5 of differentially expressed proteins, respectively. Grey indicates proteins that are not differentially expressed.

**Additional file 8:** **Figure S8.** The protein-protein interaction network of oxidative phosphorylation and redox-sensitive signalling pathways. The interaction between oxidative phosphorylation and redox-sensitive signalling pathways (sirtuin signalling pathway, AMPK signalling, actin cytoskeleton signalling, Sphingosine-1-phosphate signalling, ERK/MAPK signalling and Wnt/β-catenin signalling pathways). The sirtuin signalling pathway was the most associated signalling pathway.

**Additional file 9: Figure S9.** Sample set of TMT proteomic analysis. Three six-plex TMT sets were used for the four time point analysis (D0, D3, D7 and D14) with triplicate biological replication. A sample combined with all 12 sample was labeled with Label 130 and used as a reference sample for normalization between different sets. D0, 3, 7, 14 means samples undifferented, differentiated for 3, 7 and 14 days.

**Additional file 10: Table S1.** Details of differentially expressed proteins.

**Additional file 11: Table S2.** Details of GO enrichments of each cluster of differentially expressed proteins.

**Additional file 12: Table S3.** Details of KEGG pathway enrichments of differentially expressed proteins.

**Additional file 13: Table S4.** Details of activation prediction of canonical pathways from Ingenuity Pathway Analysis.

**Additional file 14.** Supplemental materials and methods.

## Data Availability

All data generated or analysed during this study are included in this published article.

## Abbreviations

Akt: Serine/threonine-protein kinase
ALP: Alkaline phosphatase
AMPK: Adenosine 5′-monophosphate-activated protein kinase
ANXA2: Annexin A2
ARS: Alizarin red S
ATP: Adenosine triphosphate
BP: Biological process
CC: Cellular component
CDK9: Cyclin-dependent kinase 9
COL11A1: Collagen type XI alpha 1 chain
COL14A1: Collagen type XIV alpha 1 chain
COL18A1: Collagen type XVIII alpha 1 chain
COL1A1: Collagen type I alpha 1 chain
COL1A2: Collagen type I alpha 2 chain
COL3A1: Collagen type I alpha 3 chain
COL5A1: Collagen type V alpha 1 chain
COL6A1: Collagen type VI alpha 1 chain
COL6A2: Collagen type VI alpha 2 chain
COL6A3: Collagen type VI alpha 3 chain
DEPs: Differentially expressed proteins
ECM: Extracellular matrix
ERK: Extracellular regulated protein kinases
ETC: Electron transport chain
FBLN1: Fibulin 1
FBLN5: Fibulin 5
FBN1: Fibrillin 1
FBN2: Fibrillin 2
FoxO: Forkhead box O
GO: Gene Ontology
Hdac: Histone deacetylase
HIF-1: Hypoxia-inducible factor 1
hPDLSCs: Human periodontal ligament stem cells
IPA: Ingenuity Pathway Analysis
KEGG: Kyoto Encyclopedia of Genes and Genomes
MAPK: Mitogen-activated protein kinase
MCM: Minichromosome maintenance
MF: Molecular function
MMP: Matrix metalloproteinase
mTOR: Mechanistic target of rapamycin kinase
NAD: Nicotinamide adenine dinucleotide
OXPHOS: Oxidative phosphorylation
PCNA: Proliferating cell nuclear antigen
PDGFRA: Platelet-derived growth factor receptor alpha
PI3K: Phosphatidylinositol-4,5-bisphosphate 3-kinase catalytic subunit alpha
PPI: Protein-protein interaction
ROS: Reactive oxygen species
RUNX2: RUNX family transcription factor 2
S1P: Sphingosine-1-phosphate
Sir: Silent information regulator
SIRT: Sirtuin
SOD2: Superoxide dismutase 2
SSRP1: Structure-specific recognition protein 1
TGF: Transforming growth factor
TGFBR1: Transforming growth factor beta receptor 1
TIMP: Tissue inhibitor of matrix metalloproteinase
TMT: Tandem Mass Tag
