# Supplementary figures and images for: Dynamic proteomic profiling of human periodontal ligament stem cells during osteogenic differentiation
Source: Stem Cell Res Ther. 2021 Feb 3;12:98. doi: 10.1186/s13287-020-02123-6 (PMC7860046; doi:10.1186/s13287-020-02123-6)

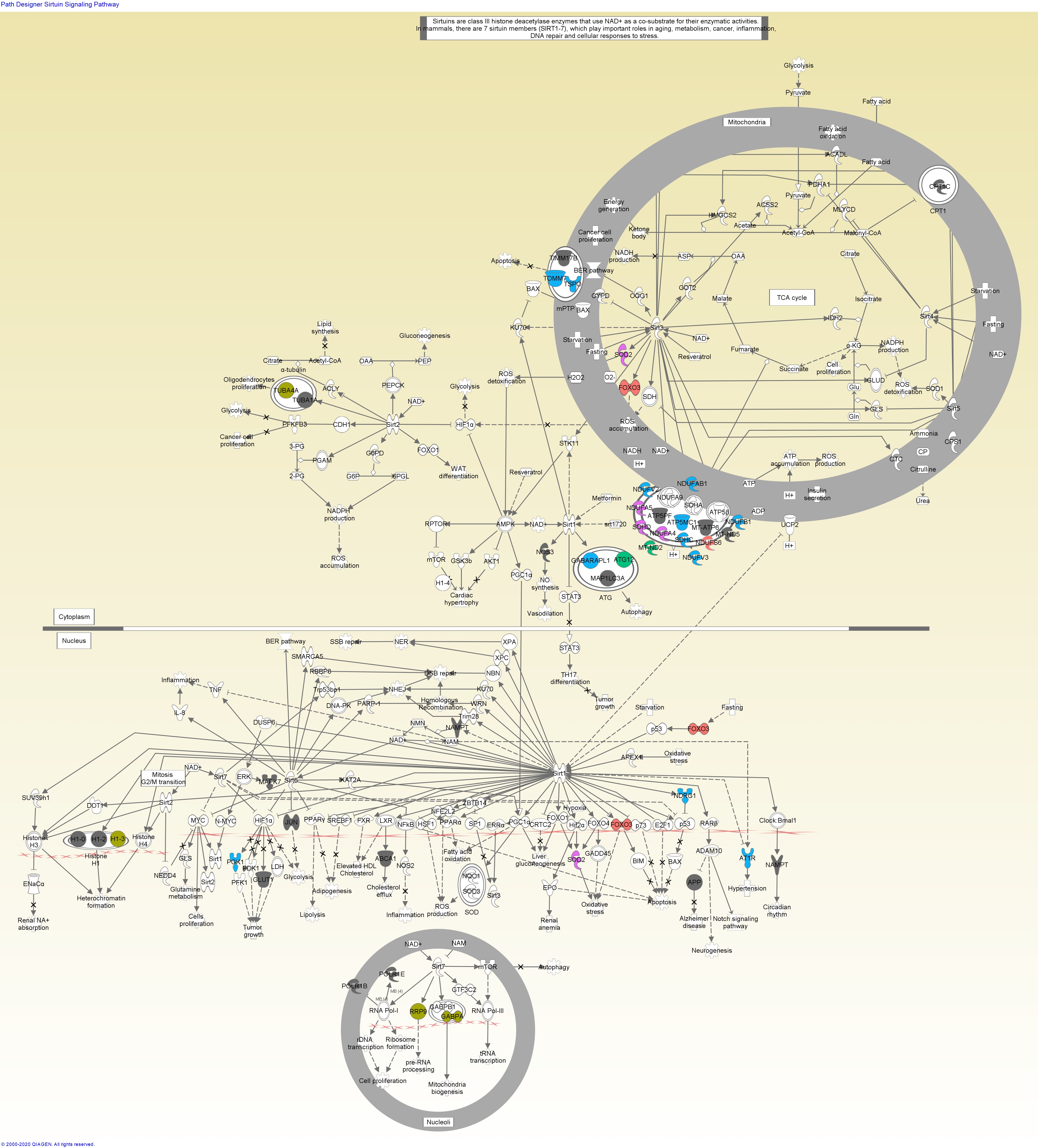

Supplement: Supplementary file 1 — Additional file 1: Figure S1. Boxplots of normalized protein ratio of all 12 samples. [file 13287_2020_2123_MOESM1_ESM.zip › Figure S1 SIRTUIN.jpg]

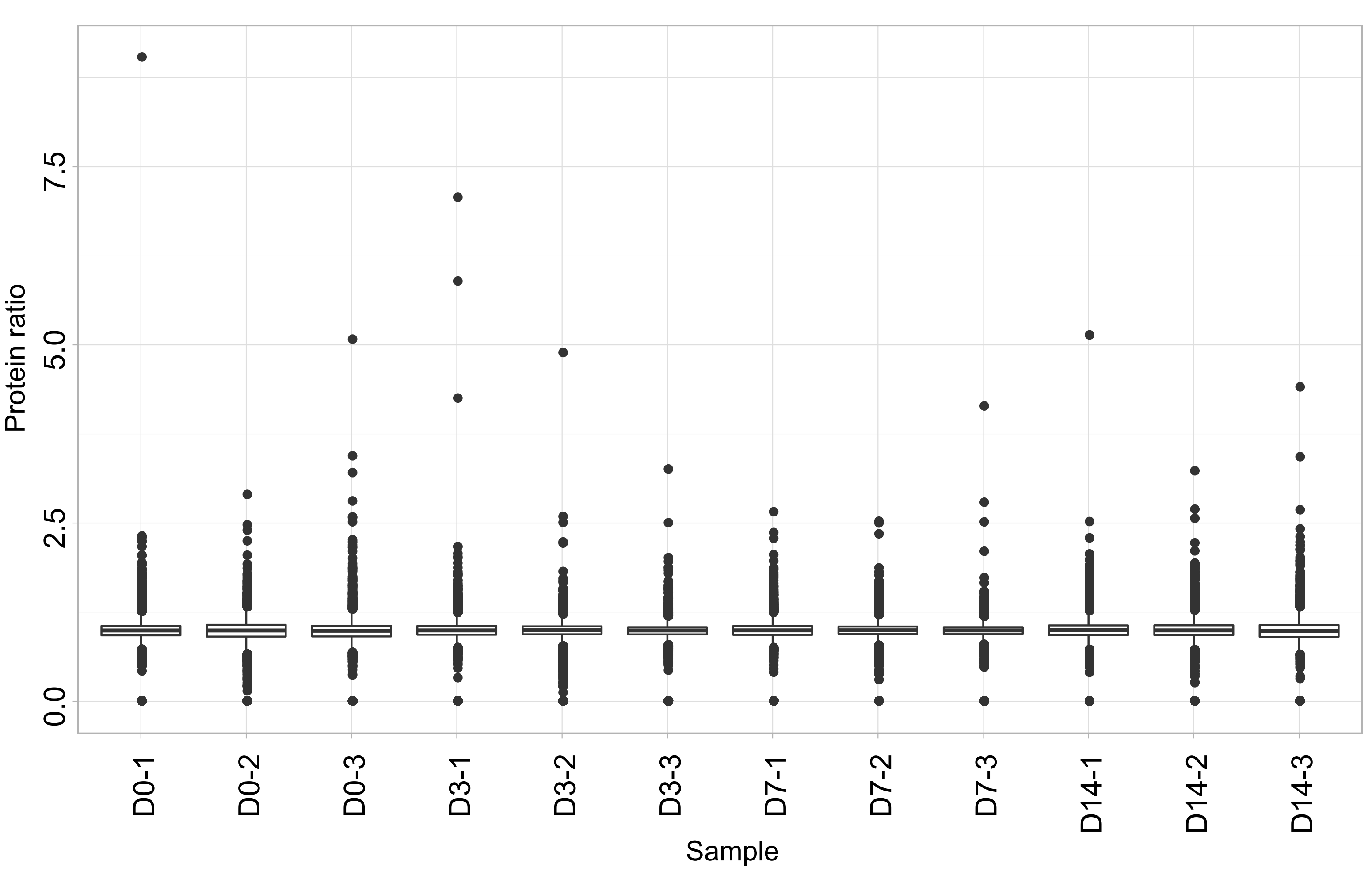

Supplement: Supplementary file 1 — Additional file 1: Figure S1. Boxplots of normalized protein ratio of all 12 samples. [file 13287_2020_2123_MOESM1_ESM.zip › Revised-Figure S1.tif]

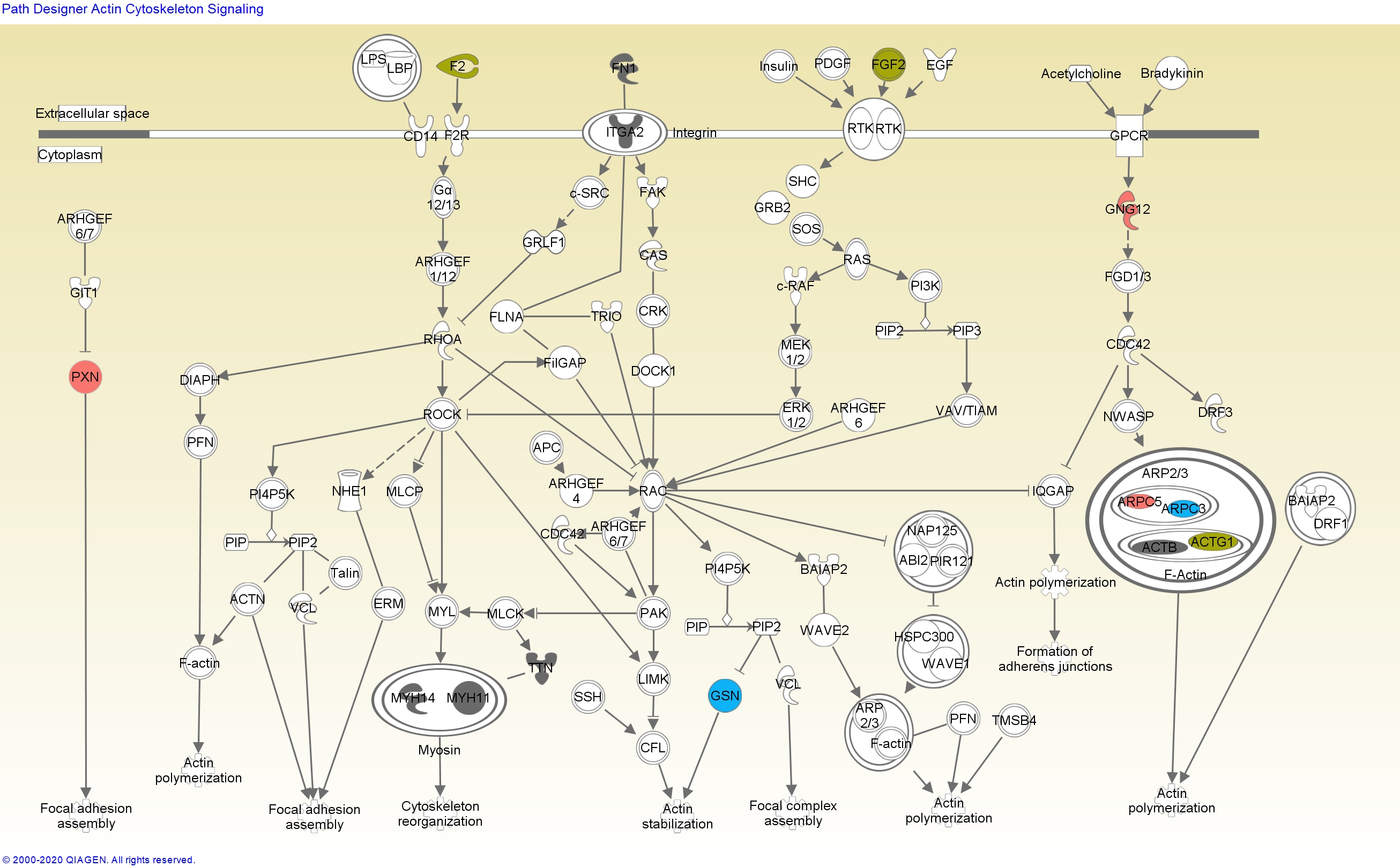

Supplement: Supplementary file 2 — Additional file 2: Figure S2. Map of the “Sirtuin signalling pathway” canonical pathway in the Ingenuity Pathway Analysis database. Proteins labelled in red, yellow, green, blue and pink are proteins from clusters 1, 2, 3, 4 and 5 of differentially expressed proteins, respectively. Grey indicates proteins that are not differentially expressed. [file 13287_2020_2123_MOESM2_ESM.jpg]

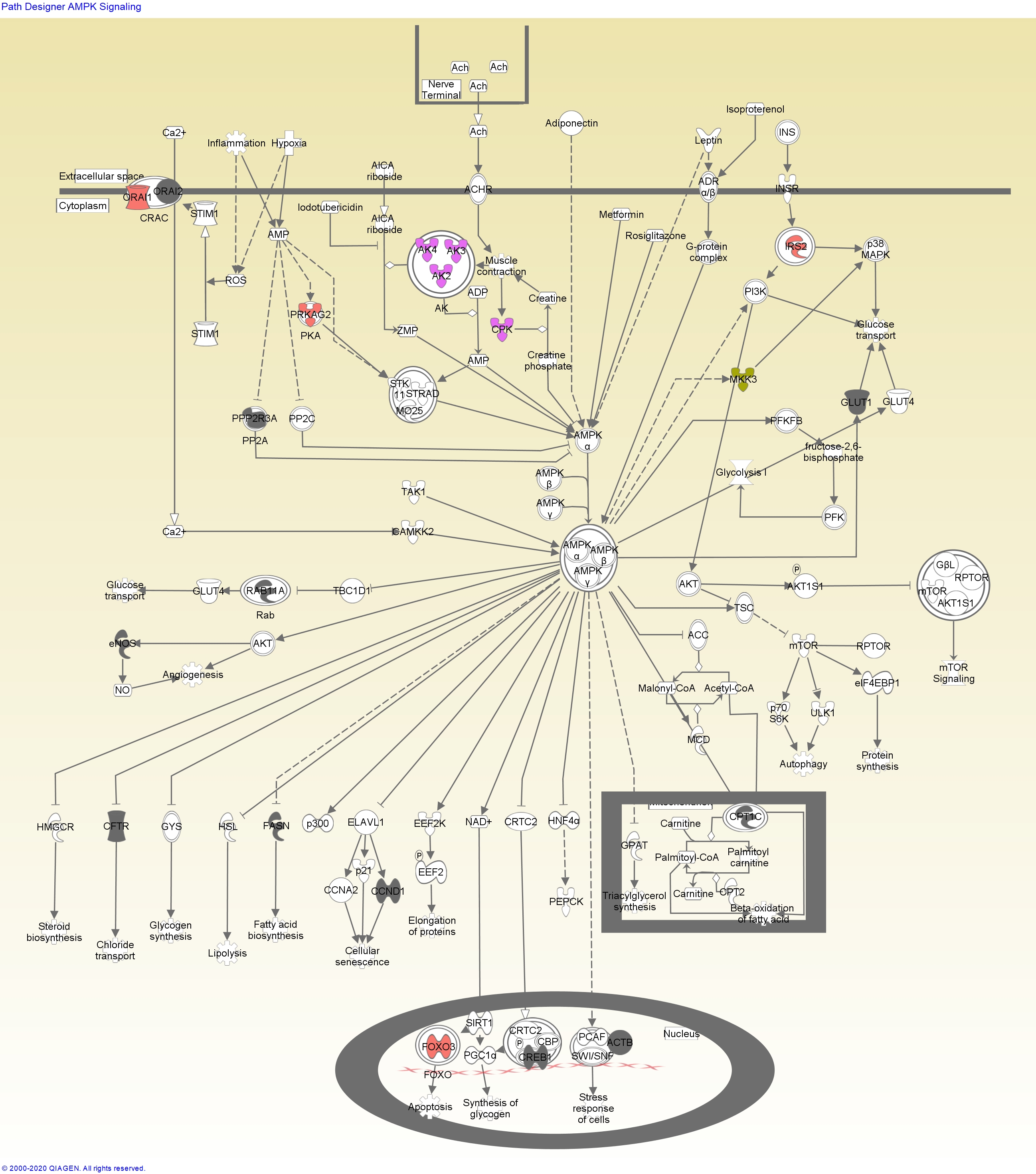

Supplement: Supplementary file 3 — Additional file 3: Figure S3. Map of the “Actin Cytoskeleton signalling pathway” canonical pathway in the Ingenuity Pathway Analysis database. Proteins labelled in red, yellow, green, blue and pink are proteins from clusters 1, 2, 3, 4 and 5 of differentially expressed proteins, respectively. Grey indicates proteins that are not differentially expressed. [file 13287_2020_2123_MOESM3_ESM.jpg]

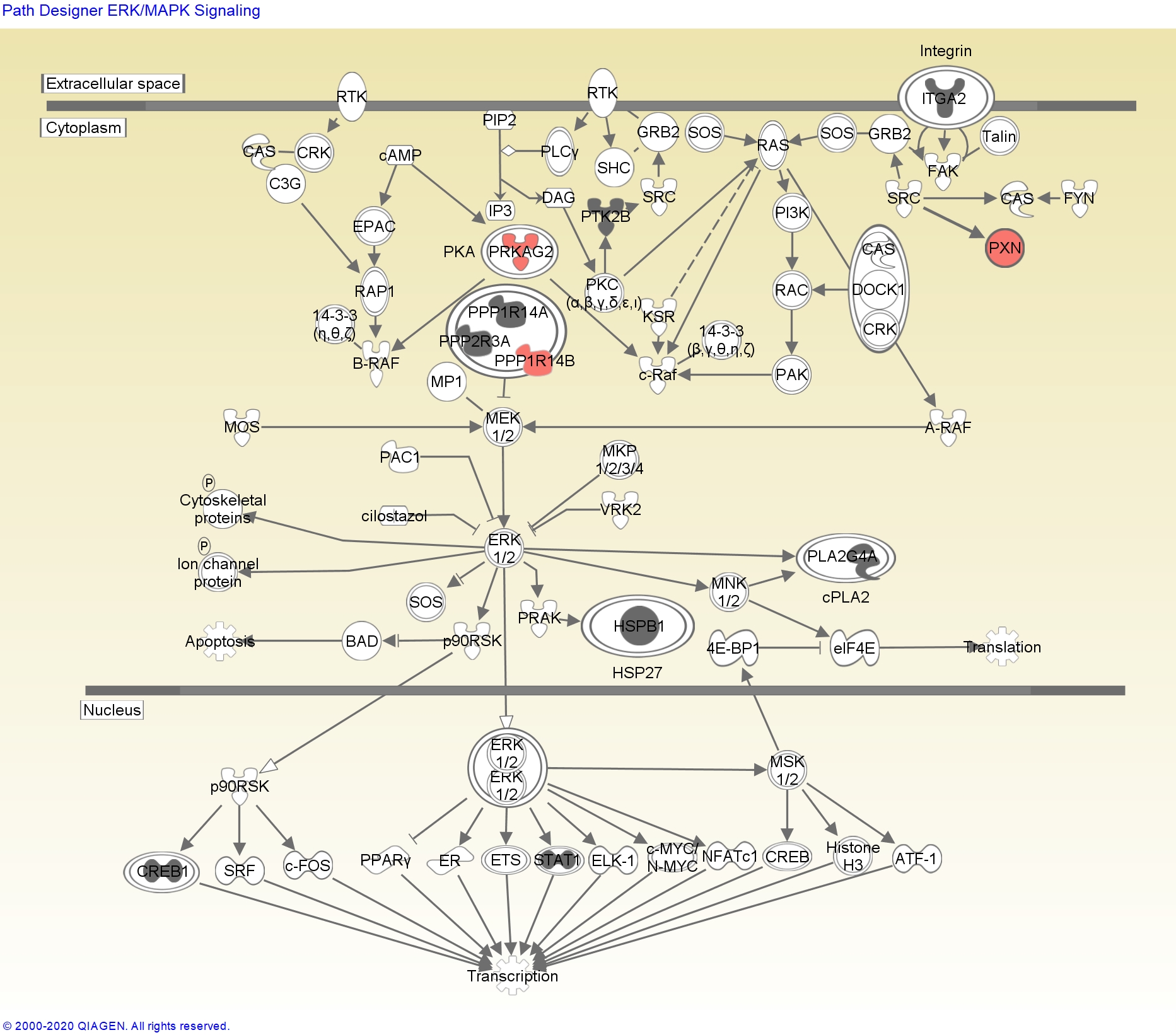

Supplement: Supplementary file 4 — Additional file 4: Figure S4. Map of the “AMPK signalling pathway” canonical pathway in the Ingenuity Pathway Analysis database. Proteins labelled in red, yellow, green, blue and pink are proteins from clusters 1, 2, 3, 4 and 5 of differentially expressed proteins, respectively. Color grey referrer to the proteins not differentially expressed. [file 13287_2020_2123_MOESM4_ESM.jpg]

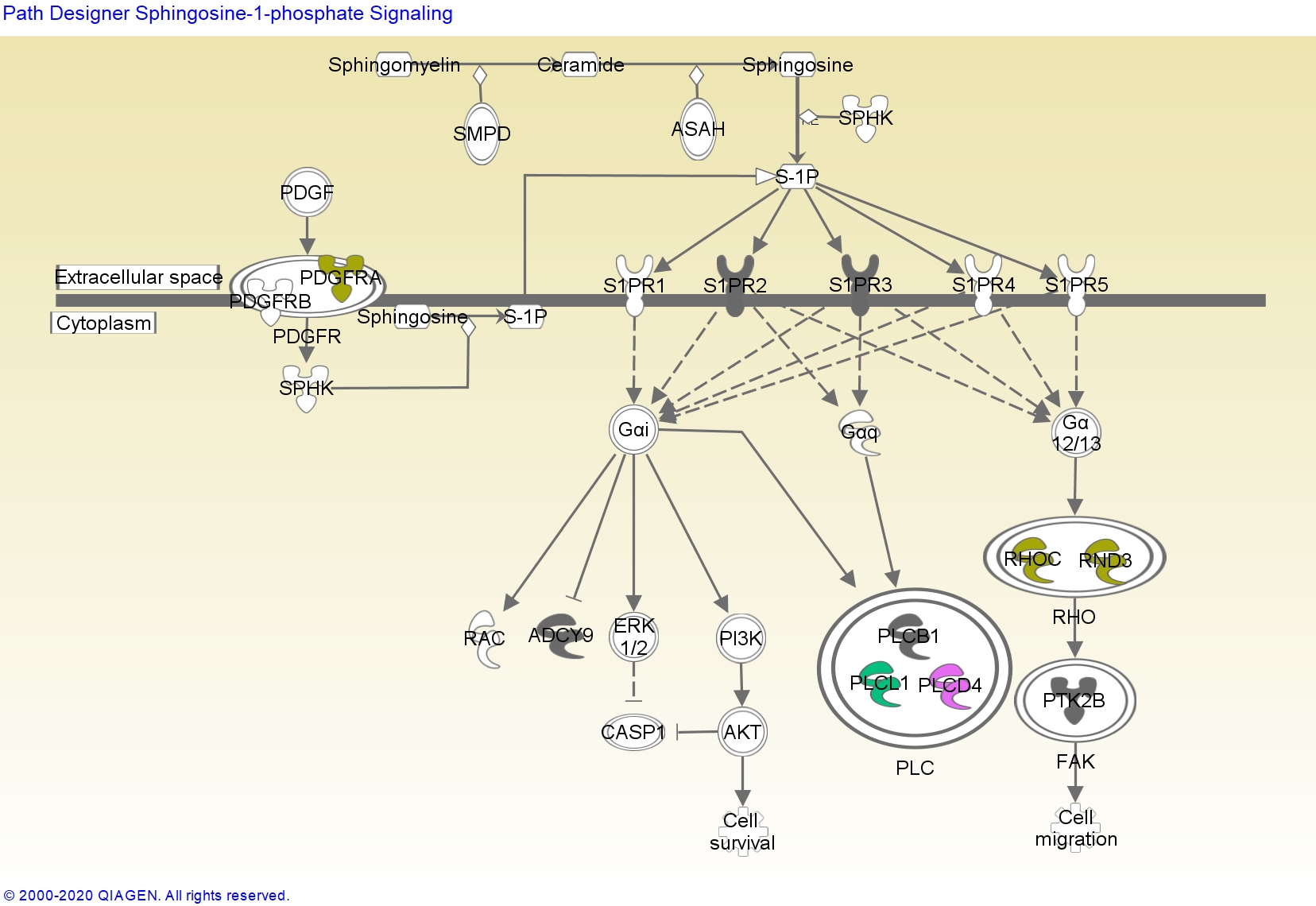

Supplement: Supplementary file 5 — Additional file 5: Figure S5. Map of “ERK/MAPK signalling pathway” canonical pathway in the Ingenuity Pathway Analysis database. Proteins labelled in red, yellow, green, blue and pink are proteins from clusters 1, 2, 3, 4 and 5 of differentially expressed proteins, respectively. Grey indicates proteins that are not differentially expressed. [file 13287_2020_2123_MOESM5_ESM.jpg]

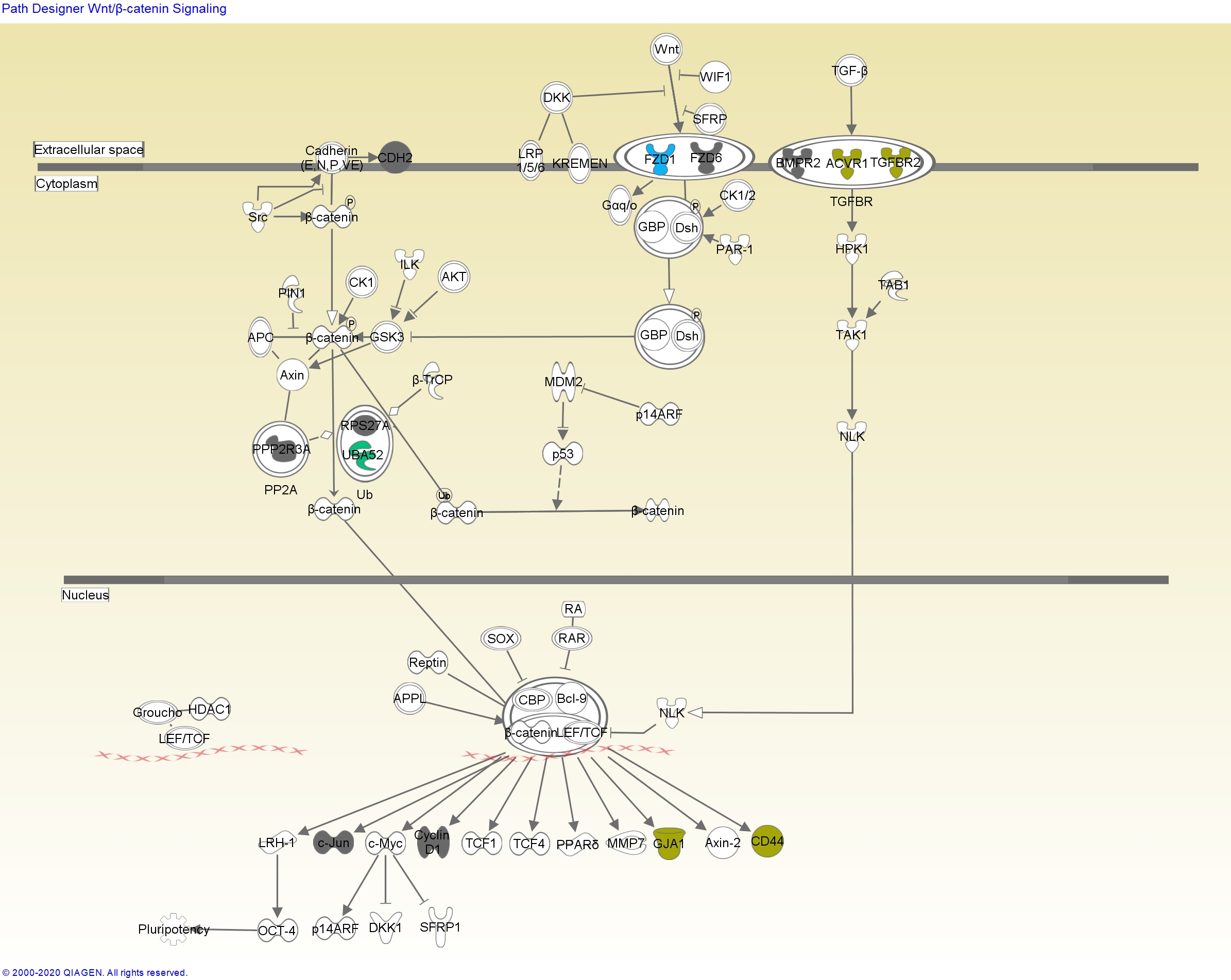

Supplement: Supplementary file 6 — Additional file 6: Figure S6. Map of the “Sphingosine-1-phosphate signalling pathway” canonical pathway in the Ingenuity Pathway Analysis database. Proteins labelled in red, yellow, green, blue and pink are proteins from clusters 1, 2, 3, 4 and 5 of differentially expressed proteins, respectively. Grey indicates proteins that are not differentially expressed. [file 13287_2020_2123_MOESM6_ESM.jpg]

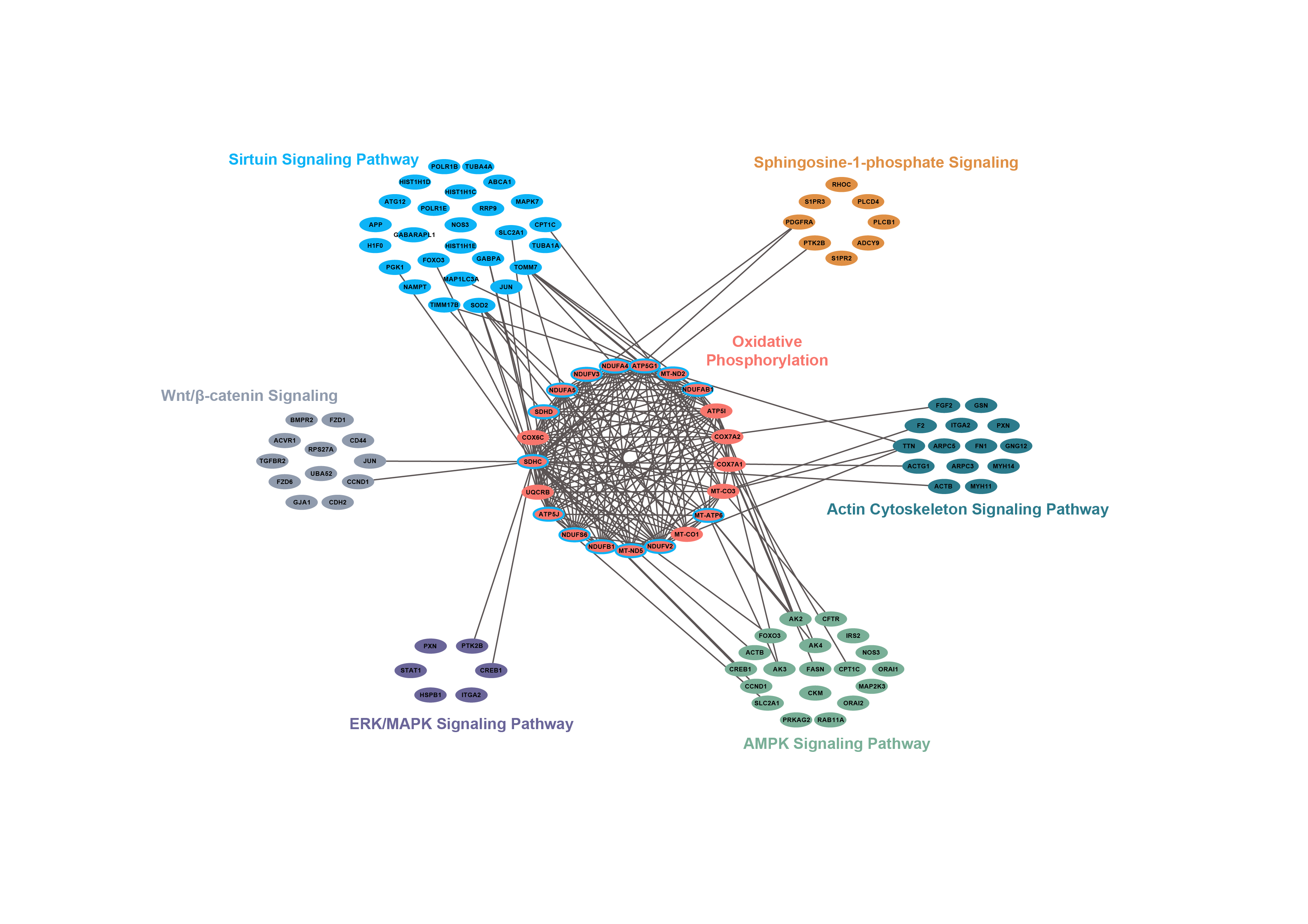

Supplement: Supplementary file 7 — Additional file 7: Figure S7. Map of the “Wnt/β-catenin signalling pathway” canonical pathway in the Ingenuity Pathway Analysis database. Proteins labelled in red, yellow, green, blue and pink are proteins from clusters 1, 2, 3, 4 and 5 of differentially expressed proteins, respectively. Grey indicates proteins that are not differentially expressed. [file 13287_2020_2123_MOESM7_ESM.tif]

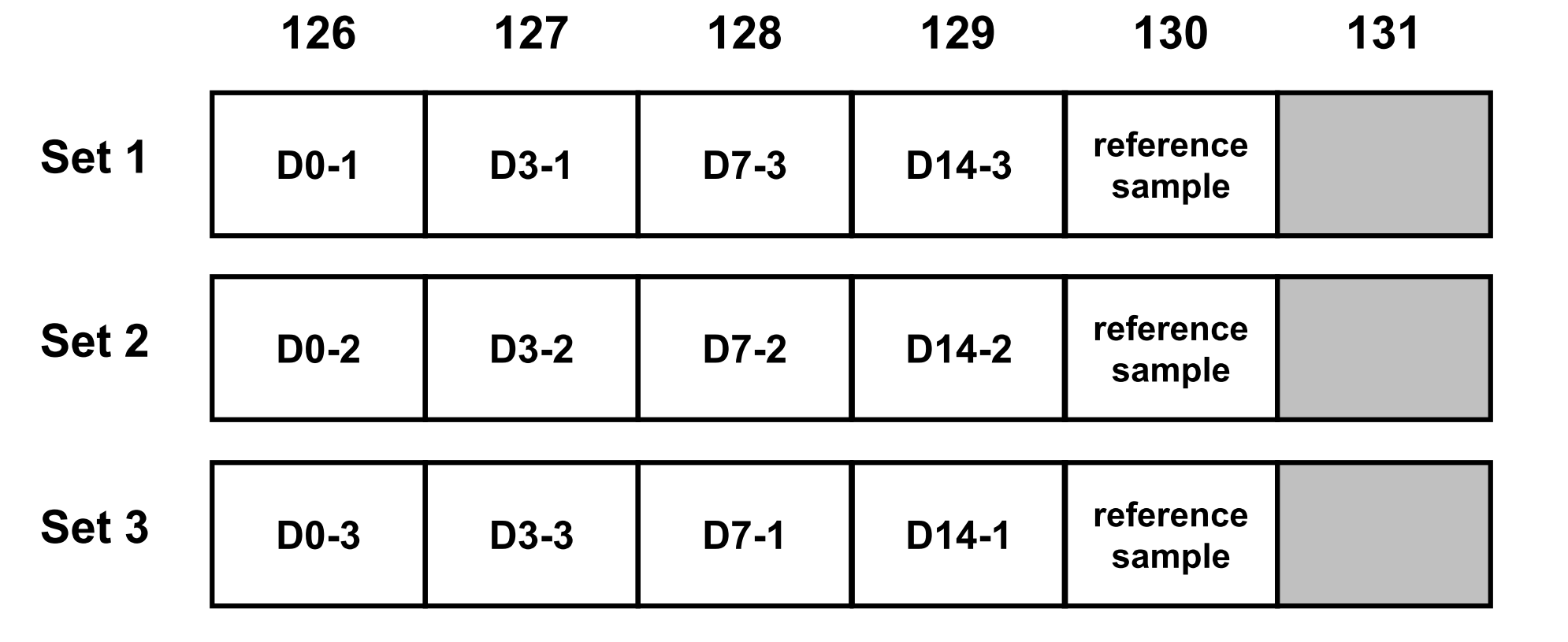

Supplement: Supplementary file 8 — Additional file 9: Figure S9. Sample set of TMT proteomic analysis. Three six-plex TMT sets were used for the four time point analysis (D0, D3, D7 and D14) with triplicate biological replication. A sample combined with all 12 sample was labeled with Label 130 and used as a reference sample for normalization between different sets. D0, 3, 7, 14 means samples undifferented, differentiated for 3, 7 and 14 days. [file 13287_2020_2123_MOESM9_ESM.tif]
