# Supplementary material for: Dynamic proteomic profiling of human periodontal ligament stem cells during osteogenic differentiation
Source: Stem Cell Res Ther. 2021 Feb 3;12:98. doi: 10.1186/s13287-020-02123-6 (PMC7860046; doi:10.1186/s13287-020-02123-6)
Supplement: Supplementary file 13 — Additional file 14. Supplemental materials and methods. [file 13287_2020_2123_MOESM14_ESM.docx]

**Supplemental MATERIALS AND METHODS**

**Alkaline phosphatase (ALP) and alizarin red S (ARS) stainings**

To verify the establishment of the osteogenesis phenotype, ALP and ARS stainings were performed on days 0, 3, 7 and 14, respectively. Cells were fixed for 30 min with 4% paraformaldehyde at 4 ℃ followed by washing with pure water three times. ALP staining was performed with an Alkaline Phosphatase Color Development Kit (Beyotime, Shanghai, China) and 1% ARS (pH=4.2) dye (Leagene, Beijing, China) for visualization of matrix mineralization.

**Cell harvest and sample preparation**

We conducted a label-dependent method to uncover the dynamic protein profiles of hPDLSCs at the undifferentiated stage and diverse differentiation stages, including days 3, 7 and 14. SDT buffer (4% SDS, 100 mM Tris/HCl, 1 mM DTT, pH=7.6) was added to the cell samples, and the lysates were boiled for 15 min. After centrifugation at 14000g for 40 min, the supernatant was quantified with BCA protein assay kit (Beyotime, Shanghai, China). The samples were stored at -80 °C until samples of all time points were collected. To ensure the qualities of the protein lysates, 20 µg of protein for each sample was separated on a 12.5% SDS-PAGE gel to check the protein bands by Coomassie Blue R-250 staining. Samples with clear bands and sufficient amounts of protein were used for the following tests.

**Protein digestion, TMT labelling and peptide fractionation**

Quantitative proteomic analysis by TMT technology was carried out at Shanghai Applied Protein Technology Company. Two hundred micrograms of protein for each sample was incorporated into 30 μL SDT buffer (4% SDS, 100 mM DTT, 150 mM Tris-HCl, pH=8.0). After the removal of detergent, DTT and other low-molecular-weight components with UA buffer (8 M urea, 150 mM Tris-HCl, pH=8.0) and repeated ultrafiltration, reduced cysteine residues were blocked using 100 μL iodoacetamide (100 mM IAA in UA buffer) for 30 min in darkness. Since the filters were washed with buffers, the protein suspensions were digested with 4 μg trypsin (Promega, Madison, WI, USA) in 40 μL TEAB buffer overnight at 37 °C, and the resulting peptides were collected as a filtrate. The peptide content was estimated by ultraviolet (UV) spectral density at 280 nm using an extinction coefficient of 1.1 of 0.1% (g/l) solution that was calculated on the basis of the frequency of tryptophan and tyrosine in vertebrate proteins. Then, 100 μg peptide of each sample was labelled using TMT-6-plex reagents (Thermo Fisher Scientific, Waltham, MA, USA). Specifically speaking, 5 labels from each kit was used to label any replicate sample of each time point along with a reference sample combined with all 12 samples (showed in Figure S9). A total of 3 pools of peptides were labelled and Pierce high pH reversed-phase fractionation kit (Thermo Fisher Scientific, Waltham, MA, USA) was used to fractionate TMT-labeled samples into 30 fractions, with 10 for each pool, by an increasing acetonitrile step-gradient elution according to instructions. In details, the dried peptide mixture of each pool was reconstituted and acidified with 0.1% TFA solution and loaded to the equilibrated, high-pH, reversed-phase fractionation spin column. A step gradient of 10 increasing acetonitrile concentrations (8%, 10%, 11.5%, 13%, 14.5%, 16%, 17.5%, 19%, 22% and 50%.) in a volatile high-pH elution solution is then applied to the columns to elute bound peptides into 10 different fractions collected by centrifugation. The collected fractions were desalted on C18 Cartridges (Empore™ SPE Cartridges C18 (standard density), bed I.D. 7 mm, volume 3 ml, Sigma) and concentrated by vacuum centrifugation.

**HPLC and MS/MS analysis**

Each fraction with approximately 1 μg was injected for nanoLC-MS/MS analysis. The peptide mixture was loaded onto a reversed-phase trap column (Thermo Fisher Scientific Acclaim PepMap100, 100 μm*2 cm, nanoViper C18) connected to the C18-reversed-phase analytical column (Thermo Fisher Scientific Easy Column, 10 cm long, 75 μm inner diameter, 3 μm resin) in buffer A (0.1% formic acid) and separated with a linear gradient of buffer B (84% acetonitrile and 0.1% formic acid) at a flow rate of 300 nL/min controlled by IntelliFlow technology. LC-MS/MS analysis was performed on a Q Exactive mass spectrometer (Thermo Scientific) that was coupled to an Easy nLC (Thermo Fisher Scientific, Waltham, MA, USA) for 60 min. The mass spectrometer was operated in positive ion mode. MS data were acquired using a data-dependent top 10 method dynamically choosing the most abundant precursor ions from the survey scan (300–1800 m/z) for high-energy collisional dissociation (HCD) fragmentation. The automatic gain control target was set to 1e6, and the maximum injection time was 50 ms. The dynamic exclusion duration was 60.0 s. Survey scans were acquired at a resolution of 70,000 at m/z 200, the resolution for HCD spectra was set to 17,500 at 200 m/z, and the isolation width was 2 m/z. The normalized collision energy was 30 eV and the underfill ratio, which specifies the minimum percentage of the target value likely to be reached at the maximum fill time, was defined as 0.1%. The instrument was run with peptide recognition mode enabled.

**Protein identification and quantitative analysis**

MS/MS spectra were searched using the Mascot engine (Matrix Science, version 2.2) embedded into Proteome Discoverer 1.4 (Thermo Fisher Scientific, Waltham, MA, USA). The Mascot parameters for protein identification were set as follows: tryptic specificity was required in all cases; 2 missed cleavages were allowed; carbamidomethylation (C), TMT 6 plex (N-terminal) and TMT 6 plex (lysine, K) were set as the fixed modifications; oxidation (methionine, M) and TMT 6 plex (tyrosine, Y) were set as the variable modifications; peptide mass tolerances were set at 20 ppm for all MS1 spectra acquired; and fragment mass tolerances were set at 0.1 Da for all MS2 spectra acquired; the peptide and protein false discovery rate (FDR) was set to ≤ 0.01. The methods for protein quantification were as followed: first, the ratio of label signals was output and calculated as a ratio to the reference sample; secondly, the necessary normalization on protein median was performed to eliminate experimental error utilizing Correcting Experimental Bias function with built-in settings in Thermo.Discoverer.UserGuide-1.4 software; finally, the protein is quantified based on the median of relative intensity of the unique peptide of each protein.

**RNA Extraction and qRT-PCR**

Total RNA of osteogenic hPDLSC samples at days 0, 3, 7 and 14 was extracted using RNA isolater Total RNA Extraction Reagent (Vazyme). cDNA was reverse-transcribed using a HiScript 1st Strand cDNA Synthesis Kit (Vazyme) followed by quantitative real-time PCR using AceQ Universal SYBR qPCR Master Mix (Vazyme). GAPDH was used as an internal control. The primer sequences were as follows: GAPDH, 5'-AACGGATTTGGTCGTATTGGG-3' (forward) and 5'-CCTGGAAGATGGTGATGGGAT-3' (reverse); SOD2, 5'-GCTGGAAGCCATCAAACGTG-3' (forward) and 5'-GAAACCAAGCCAACCCCAAC-3' (reverse). The experiments were performed at least in triplicate. The relative gene expression analysis and fold change were calculated using the 2^−ΔΔCt^ method.

**Western Blotting Analysis**

The hPDLSCs undergoing osteogenic differentiation for the indicated times were lysed in 1X RIPA buffer (Beyotime) containing 1X protease inhibitor cocktail (Meilunbio) at the final concentrations. Cell debris was removed, and the clear supernatant was collected after centrifugation at 12,000× g for 20 min at 4 °C. Protein concentration was determined using a BCA protein assay kit (Beyotime). Total lysates (20-40 μg) were loaded onto 8%, 10% or 12% SDS-PAGE gels, and the gel was transferred onto a 0.45 μm polyvinylidene difluoride membrane (Millipore) at 250 mA for 1-4 h at 4 °C. The membrane was blocked with 5% skim milk dissolved in Tris-buffered saline containing 0.05% Tween 20 and probed with various primary antibodies at the indicated dilutions (RUNX2, ALP, FBN1, SOD2, OXPHOS complex, GAPDH) overnight at 4 °C. The membrane was then incubated with secondary antibodies (anti-rabbit, anti-mouse, 1:10,000) and detected by the enhanced chemiluminescence reagents (Biosharp Life Sciences) on an image analyser. The levels of target proteins were normalized to GAPDH, which served as a reference control. The intensity of protein bands was analysed with ImageJ Software and values are expressed as the means ± standard deviation (SD) of at least three independent experiments.
